# The role of life history traits in mammalian invasion success

**DOI:** 10.1111/ele.12493

**Published:** 2015-08-21

**Authors:** Isabella Capellini, Joanna Baker, William L. Allen, Sally E. Street, Chris Venditti

**Affiliations:** ^1^ School of BiologicalBiomedical and Environmental SciencesUniversity of HullCottingham RoadHullHU6 7RXUK; ^2^ School of Biological SciencesUniversity of ReadingReadingRG6 6BXUK

**Keywords:** Alien species, biological invasions, colonisation success, demography, invasion pathway, life history theory, mammals, phylogeny, propagule pressure, range expansion

## Abstract

Why some organisms become invasive when introduced into novel regions while others fail to even establish is a fundamental question in ecology. Barriers to success are expected to filter species at each stage along the invasion pathway. No study to date, however, has investigated how species traits associate with success from introduction to spread at a large spatial scale in any group. Using the largest data set of mammalian introductions at the global scale and recently developed phylogenetic comparative methods, we show that human‐mediated introductions considerably bias which species have the opportunity to become invasive, as highly productive mammals with longer reproductive lifespans are far more likely to be introduced. Subsequently, greater reproductive output and higher introduction effort are associated with success at both the establishment and spread stages. High productivity thus supports population growth and invasion success, with barriers at each invasion stage filtering species with progressively greater fecundity.

## Introduction

**Figure 1:**
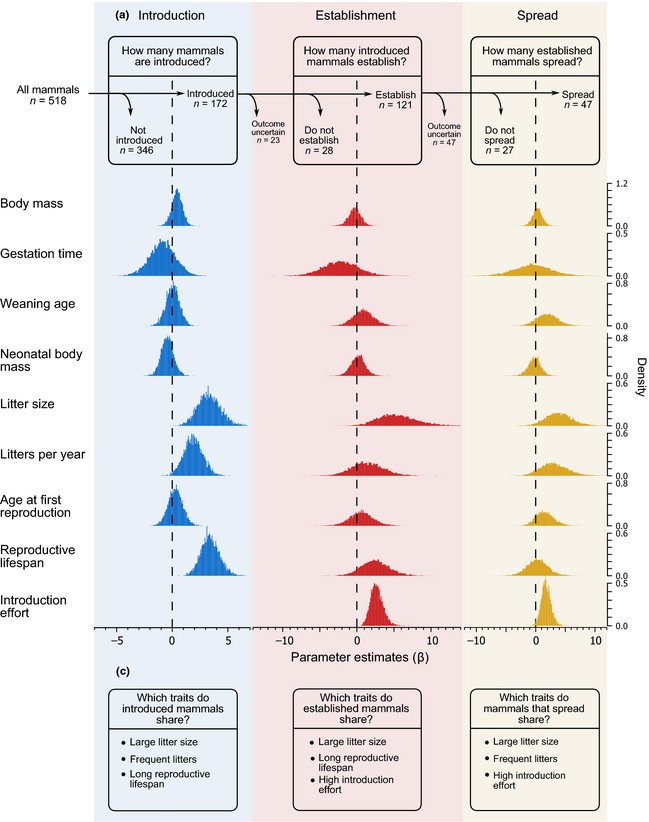
Stages of the invasion pathway and mammalian invasion success. (a) At each stage alien populations face specific barriers (vertical lines) that they need to overcome; these barriers filter species from one stage to the next (arrows). The sample sizes of successful and unsuccessful species at each invasion stage are indicated. Species for which no reliable information is available on success at establishment and spread are excluded from the analysis at that stage and subsequent stage (Supporting Information Section 1.1.1). (b) Posterior distributions of the parameter estimates (β) of life history traits and introduction effort at each stage of invasion. The posterior distribution of an independent variable with a negligible effect on the probability of success is expected to be centred on zero (dotted line); conversely the distribution of an influential variable is expected to be substantially shifted from 0. (c) Summary of the results.

The outcome of introductions of alien organisms in novel environments varies greatly across species. Following release into a non‐native environment (introduction), an alien population establishes if it reaches a sufficient size to be self‐sustaining (establishment) but ‘invasive’ species are those that show a dramatic increase in range size post‐establishment (spread; Fig. 1a) (Kolar & Lodge 2001; Blackburn *et al*. 2011; Richardson & Pyšek 2012). Crucially, not all introduced populations establish, and not all those that establish become invasive (Kolar & Lodge 2001; Lockwood *et al*. 2005; Sol 2007; Blackburn *et al*. 2011; Richardson & Pyšek 2012). Such diversity in the success of alien organisms depends on the interplay between the characteristics of the introduced species, the receiving community and the peculiarities of the introduction event (Kolar & Lodge 2001; Lockwood *et al*. 2005; Sol 2007; Blackburn *et al*. 2011; Richardson & Pyšek 2012). Here, we focus on the characteristics of the alien species and ask which life history traits promote success from introduction through to spread, as predicted by two contrasting theoretical models of population growth.

Recent ecological frameworks view biological invasions as a series of sequential stages along an invasion pathway (introduction, establishment, spread), each characterised by specific barriers that alien populations need to overcome (Kolar & Lodge 2001; Lockwood *et al*. 2005; Blackburn *et al*. 2011; Richardson & Pyšek 2012). Because organisms may face different challenges across stages, factors promoting success at one stage might be irrelevant or even detrimental at the next (Kolar & Lodge 2001; Cassey *et al*. 2004b; Blackburn *et al*. 2011; Richardson & Pyšek 2012). Therefore, investigating the invasion pathway in its entirety is essential to fully unravel how species' traits contribute to success in novel regions. Surprisingly, how barriers filter species from introduction to spread has not been investigated at large scales, as no study to date has considered more than one or two invasion stages. Specifically, we currently have a very limited understanding of which species traits increase the likelihood of spread in animals at the global scale since most studies have focused exclusively on establishment (Kolar & Lodge 2001; Sol 2007). Due to the sequential nature of biological invasions, however, the determinants of success at establishment may be little informative for understanding what promotes success at spread (Richardson & Pyšek 2012). Likewise, merging stages together (e.g. Jeschke & Strayer 2006; Burns *et al*. 2011; Gibson *et al*. 2011) can mask potential associations between species traits and success, and ultimately lead to erroneous conclusions (Cassey *et al*. 2004a; van Kleunen *et al*. 2010a). The stage of introduction, and specifically whether the sample of introduced species share particular characteristics (‘introduction bias’), is also rarely considered in animal studies at small geographical scales and in plant studies at the global scale (van Kleunen *et al*. 2010a; Richardson & Pyšek 2012). This is problematic because barriers to transport and human preferences filter species for introduction, resulting in a non‐random sample of introduced species and an uneven opportunity, across species within a taxon, to express their potential to be invasive (Blackburn & Duncan 2001; Cassey *et al*. 2004b; Jeschke & Strayer 2006; Blackburn *et al*. 2011; Hui *et al*. 2011; Richardson & Pyšek 2012). Introduction biases can potentially enhance the chances of invasion, if organisms with characteristics facilitating success in the novel environment are accidentally or intentionally selected (Blackburn & Duncan 2001; Cassey *et al*. 2004b; Jeschke & Strayer 2006; Blackburn *et al*. 2011). Therefore, possible introduction biases need to be identified if we are to build a comprehensive picture of how factors, such as species' traits, contribute to success at later invasion stages.

The magnitude of introduction effort (also called ‘propagule pressure’; Lockwood *et al*. 2005) is one of the major predictors of success of non‐native populations, as alien organisms are typically introduced in small numbers (Lockwood *et al*. 2005; Simberloff 2009). Theoretical models of extinction risk in founder populations and empirical evidence show that the likelihood of establishing increases with the number of introduced individuals (Cassey *et al*. 2004c; Lockwood *et al*. 2005; Simberloff 2009; Richardson & Pyšek 2012; Sol *et al*. 2012; Blackburn *et al*. 2015). Larger populations have greater chances to escape extinction due to demographic and environmental stochasticity (e.g. random fluctuations in sex ratios or unusual weather conditions; Pimm 1991; Simberloff 2009), are less likely to suffer from inbreeding depression, genetic bottlenecks and drift (Pimm 1991; Simberloff 2009; Forsman 2015) and, in some species, Allee effects (reduced fitness at low population density; Simberloff 2009). Nonetheless, considerable unexplained variation in establishment success remains after accounting for initial population size. Life history traits are central in this context as they shape key demographic parameters and determine how founder populations grow beyond the size at which the risk of extinction is small (Sol 2007; Gurevitch *et al*. 2011; Blackburn *et al*. 2015). The same life history traits that determine the initial population growth should also facilitate success at the stage of spread, since introduced populations that successfully establish and colonise large areas must increase in size at both these stages (Lockwood *et al*. 2005; Sol 2007; Blackburn *et al*. 2011, 2015; Gurevitch *et al*. 2011). However, how exactly life history traits support population growth is debated.

Classic theory suggests that ‘fast’ life histories – such as frequent reproductive events, high fecundity, early maturity – promote rapid population growth, resulting in a minimum population size that evades the risk of extinction quickly (Pimm 1991). In support of this hypothesis, earlier reproducing conifers (class Pinopsida) (Richardson & Rejmanek 2004), reptiles and amphibians (van Wilgen & Richardson 2012), succeed better in establishing alien populations than species with a slower pace of life. Fast life histories, however, are unrelated to establishment success in birds (Cassey *et al*. 2004b; Blackburn *et al*. 2009; Sol *et al*. 2012) and mammals (Sol *et al*. 2008).

Because the colonisation of large areas requires that the established alien populations increase further in size (Lockwood *et al*. 2005; Blackburn *et al*. 2011; Richardson & Pyšek 2012), the predictions of theoretical models on how life history traits support population growth should equally apply to the stage of spread (Sol 2007; Blackburn *et al*. 2015). Studies at small geographical scale on success at spread are, however, contradictory, finding support for classic theory in mammals (Forsyth *et al*. 2004) and plants (Hamilton *et al*. 2005; van Kleunen *et al*. 2010b), but not in birds (Duncan *et al*. 2001) and fish (Marchetti *et al*. 2004).

Contrary to classic theory (Pimm 1991), a recent theoretical model (Sæther *et al*. 2004) proposes that fast reproducing species are more (not less) susceptible to extinction at low population sizes because they are also short‐lived. Conversely, long‐lived species may adopt a more flexible strategy in variable environments, by postponing reproduction in bad years and investing in larger clutches or litters in good years (‘bet‐hedging’; Seger & Brockmann 1987). The ‘offspring value’ index (OV), used to test this model, estimates the relative fitness value for the parents of a litter or clutch relative to the species' reproductive lifespan, with low offspring values indicating that the reproductive investment is divided into several attempts across the reproductive lifespan. In support of a recent theoretical model (Sæther *et al*. 2004), birds with low OV have greater establishment success in non‐native regions (Sol *et al*. 2012). Thus, this model has the potential to help explain success at establishment and spread in other taxonomic groups, such as mammals, for which the role of life history traits in biological invasions remains elusive.

While past studies provide at least partial support for a role of life history traits in success at establishment and spread, no comparative study to date in any taxonomic group has quantified the relative contribution of multiple life history traits to success at each stage along the invasion pathway in a single taxon, and often ignore the well‐established patterns of correlated evolution among life history traits. Moreover, because statistical models for binary dependent variables (success at a stage) that incorporate phylogeny have not been available until very recently, the effect of species' shared ancestry has either been ignored and so likely underestimated (including studies using taxonomy as a proxy for phylogeny), or assumed high and possibly overestimated (e.g. independent contrasts). In addition, analyses at small geographical scales are less suited to identify generality of patterns, as they typically have small sample sizes and are more likely influenced by local factors. As a result, the extent to which barriers filter species for success at early stages with respect to life history traits and how this in turn impacts on success at later stages is still unclear.

Here, we take advantage of recently developed comparative approaches that for the first time allow testing models with binary dependent variables in a phylogenetic context (Hadfield 2010), and investigate how barriers select successful species from introduction to spread at the global scale in mammals. Using these models, we quantify the relative contribution of each life history trait to success as predicted by two opposing theoretical frameworks (Pimm 1991; Sæther *et al*. 2004).

## Methods

### Data collection

Here, we ask whether successful ‘invaders’ share life history traits that promote their success when introduced into novel environments. As recommended by van Kleunen *et al*. (2010a), to answer this question we compare traits of successful and unsuccessful species at each stage of the invasion pathway (Fig. 1a). For later stages (establishment, spread) we use only species that passed successfully the previous stage (Cassey *et al*. 2004a; van Kleunen *et al*. 2010a). Therefore, to investigate the determinants of success at establishment, we consider only those species that have been introduced (and discard those that have never been introduced); likewise for spread we use the subset of species that have successfully established (and do not include in this analysis those that have failed to establish) (Fig. 1a).

We extract data on the status of alien mammals at the global scale by integrating, cross‐checking and updating available information from three main sources (Long 2003; DAISIE 2008; IUCN 2013) complemented with more recent sources (Section 4.2 of the Supporting Information). We thus build the most comprehensive data set of mammalian introductions at the global scale to date (species with invasion data, i.e. introduced/alien: *n* = 232; species not introduced: *n* = 3458). The full Supporting Information is available from the Dryad Digital Repository: doi:10.5061/dryad.rk4jp.

We design a protocol for the classification and status of alien mammals that is conservative in that we discard all unconfirmed records, all records for which we do not find sufficient information or for which the available information is ambiguous (Supporting Information Section 1.1.1). At least two of the authors double‐checked all ambiguous records, all the records from introduction to spread for 10% randomly selected species classified as introduced, and all the critical records for the outcome at spread for all successfully established species (Supporting Information Section 1.1.1).

We are interested in whether successful invaders share life history traits that promote their success in novel regions, rather than in the location characteristics that facilitate invasions. Studies on success of individual introductions at small geographical scales show that success at a stage in one location is strongly predicted by success of the same species at other locations; species identity is a very strong predictor of success; and failures at one location of otherwise successful species is primarily explained by introduction to unsuitable habitats and low introduction effort (Supporting Information Section 1.1.2). On the basis of this evidence, we consider one successful event at a stage as sufficient evidence that the species has the potential to succeed at that stage and therefore we classify species as successful at a given stage if they have succeeded at that stage at least once (Supporting Information Sections 1.1.1 & 1.1.2). Specifically, we regard as alien a species that is introduced intentionally or accidentally by humans outside its native range. A successfully established species is one for which at least one of its alien populations persists in the novel habitat for a time interval equal or greater than the species' maximum lifespan. This temporal interval ensures that an introduced population has had sufficient time to establish and is applicable also to cases where an alien population is recorded as ‘still present’ or ‘established’ without any specific mention of successful reproduction. We use the longest recorded lifespan as reported in PanTHERIA (Jones *et al*. 2009), AnAge (De Magalhaes & Costa 2009), Ernest (2003) and Carey & Judge (2000), or alternative sources if unavailable from these references (Supporting Information Section 4.2). Among the established species, we consider a species successful at spread if at least one of its established populations exhibits a remarkable range expansion beyond the introduction location (full details for all stages in Supporting Information Section 1.1.1; species sample sizes in Fig. 1a).

Species introduced in large numbers and to more unique locations have greater opportunities to overcome extinction risk and have an increased probability of being introduced into an ecologically appropriate environment (Duncan *et al*. 2001; Forsyth *et al*. 2004; Lockwood *et al*. 2005; Sol *et al*. 2012). The number of introduced individuals, introduction events per locality, and unique introduction locations are positively associated with one another in studies across species in mammals and birds (e.g. Duncan *et al*. 2001; Forsyth & Duncan 2001; Cassey *et al*. 2004c; Forsyth *et al*. 2004). The number of introduced individuals is also strongly associated with the number of introduction locations in our data set (Supporting Information Section 1.1.3). Moreover, when tested together, the number of unique introduction locations is an equal or better predictor of success of alien mammals and birds introduced to Australia (Duncan *et al*. 2001; Forsyth *et al*. 2004). Therefore, to maximise sample sizes, we follow previous studies (Duncan *et al*. 2001; Forsyth & Duncan 2001; Forsyth *et al*. 2004; Krivánek *et al*. 2006) and use the number of unique locations each species has been introduced to as an estimate of the magnitude of introduction effort (Supporting Information Section 1.1.3). Information on the number of introduction locations is available from the same sources used for the classification of alien mammals (Long 2003; DAISIE 2008; IUCN 2013; Supporting Information Section 4.2).

We limit the main analysis to the subset of species for which all life history traits to be tested are also available (total *n* = 518 species, of which 172 introduced; Fig. 1a; Supporting Information Section 1.1.1). We extract life history traits from the PanTHERIA database (Jones *et al*. 2009) and complement this data set with data from Ernest (2003) and more recent sources for missing values (Supporting Information Section 4.2). We consider the following life history traits: gestation time (GT, days), weaning age (WA, days), age at first birth (AFB, days), litter size, number of litters per year (LY), neonatal body mass (NBM, g), adult body mass (ABM, g) and reproductive lifespan (RL, days). Where interbirth interval (days) is reported instead of litters per year, we convert interbirth interval into number of litters per year. Reproductive lifespan is calculated as the difference between maximum lifespan and age at first birth. Lifespan data for all species are taken from the same sources used to evaluate establishment success (Carey & Judge 2000; Ernest 2003; De Magalhaes & Costa 2009; Jones *et al*. 2009; Supporting Information Section 1.1.1). We calculate an ‘offspring value’ index as in previous studies (Sol *et al*. 2012; Supporting Information Section 1.1.4) to test the predictions of recently proposed theoretical models (Sæther *et al*. 2004).

### Phylogenetic comparative analysis

For each stage of the invasion pathway, success at a stage is converted into a binary variable (success coded as 1, failure as 0) and entered as the response variable in all models. Life history traits and introduction effort are entered as independent variables. All life history traits are log_10_‐transformed. Introduction effort in our data set is highly skewed since the majority of species have been introduced only to one location; no transformation can normalise the distribution of this variable. We thus convert introduction effort into a binary trait by splitting the data at the sample median of the subset of all introduced species with information on establishment success (4 locations) such that 4 or fewer introduction locations are recorded as 0, and 5 or more locations as 1. Results are qualitatively similar to those presented here with very few minor exceptions, when: (1) continuous raw data or (2) log_10_‐transformed data are used, and when (3) introduction effort is converted into a binary trait using other thresholds (Supporting Information Sections 1.1.3, 2.4). In the Supporting Information, we also demonstrate that our analysis is robust and our conclusions are unaffected by potential multicollinearity among predictors and potential sampling biases (Supporting Information Sections 1.2.2, 1.2.4, 2.1, 2.3).

At each invasion stage, we model the probability of success as a function of life histories and introduction effort using phylogenetic generalised linear mixed models in a Bayesian framework (Hadfield 2010). We use a probit model in MCMCglmm (Hadfield 2010), with success/failure at a stage as binary response variable, largely uninformative priors (normal distribution with a mean of zero and a variance of 10^8^) for all predictors treated as fixed factors, and a chi‐squared prior for the phylogeny treated as a random factor as this best approximates a uniform distribution (Hadfield 2010; de Villemereuil *et al*. 2012). Because binary response variables do not provide sufficient information for estimating the residual variance, the residual variance is commonly fixed to a given value, i.e. 1 (Hadfield 2010; de Villemereuil *et al*. 2012). The MCMC chains are run for 15 million iterations with a burnin of 1 million. Model convergence and mixing are assessed by ensuring that the effective sample sizes for all estimated parameters are > 1000. We use the phylogeny of Fritz *et al*. (2009) as this is the most comprehensive tree available for mammals.

We assess the relative contribution of each independent variable to the probability of success by examining the proportion of the posterior distribution of the parameter estimates (β) crossing zero. If a variable has a negligible effect we expect its posterior distribution to be centred on zero; conversely the distribution of an influential variable is expected to be shifted from and not substantially overlapping 0. We compute average partial effects (APEs) as a measure of the effect size of the life history traits (Long 1997; Greene 2012) that have been identified as influential in the analysis. APEs are estimates of the probability of change in the response variable (from 0 to 1 and *vice versa*) for a unit change in a given independent variable, averaged across all observed values of all independent variables in the model (Long 1997; Greene 2012; Supporting Information Section 1.2.1). We derive the posterior distributions of APEs for influential life history traits at a stage from the posterior distributions of their β estimates (Greene 2012; Supporting Information Section 1.2.1).

We estimate the importance of species' shared evolutionary history using heritability (*h* ^2^), a measure of phylogenetic signal ranging between 0 and 1, that can be calculated from the estimated phylogenetic variance in the model (Hadfield 2010). The interpretation of *h* ^2^ is identical to that of λ in phylogenetic generalised least squares models (Freckleton *et al*. 2002), such that values close to 0 indicate that there is negligible effect of phylogeny, and values close to 1 that there is strong phylogenetic signal in the data. We calculate *h* ^2^ across the entire posterior distribution of model variances.

## Results

We first investigate whether introduced mammals are a random sample or whether there are biases with regard to life histories in the pool of introduced species (total species sample *n* = 518, Fig. 1a). We find that the probability of being introduced is associated with larger and more frequent litters (0.0 and 1.0% of the posterior distribution crossing zero respectively) and longer reproductive lifespan (0.0%, Fig. 1b). Thus, a day increase in reproductive lifespan corresponds to a 3.7% relative increase in the probability a species is introduced, while each additional offspring per litter and one more litter per year increase the relative probability by 3.6 and 2.1% respectively.

Next we test the predictions of classic theory and a recent theoretical model on how life history traits support population growth, and ultimately influence success at establishment and spread. Contrary to previous findings (Sol *et al*. 2008), establishment success among introduced mammals (*n* = 149, Fig. 1a) is associated with larger litters (0.9% of the posterior distribution across zero), marginally longer reproductive lifespan (8.9%) and greater introduction effort (0.0%; Fig. 1b), such that every additional offspring increases the relative probability of establishment by 3.8% and 1 day longer reproductive lifespan by 1.8%.

Successful spread among established mammals (*n* = 74, Fig. 1a) is associated with higher introduction effort (1.0% of the posterior distribution crosses zero), larger litters (3.7%) and a weaker effect of more frequent litters (8.2%, Fig. 1b). This translates into 4.9 and 3.8% greater chance of success at this stage for every additional offspring produced per litter and reproductive event per year.

Contrary to the predictions of recent theoretical models (Sæther *et al*. 2004), mammals dividing reproductive effort into more attempts (low OV, following Sol *et al*. 2012), however, have no greater probability of establishing or spreading into non‐native regions (Table 1). We also find that alien mammals are not a random sample with regard to OV, having lower OV than mammals that are not introduced into novel environments (Table 1). However, we suggest that this reflects the association between OV and life history traits in mammals (Supporting Information Sections 1.2.3, 2.2), in particular litter size, litters per year and reproductive lifespan, that are strong predictors of success at introduction.

**Table 1:** Effect of offspring value (OV) on the probability of success at introduction, establishment and spread

Stage	Introduction			Establishment			Spread		
Stats	Mean β	SD β	% β	Mean β	SD β	% β	Mean β	SD β	% β
BM	−0.04	0.13	37.2	−0.39	0.25	4.7	−0.06	0.28	39.5
OV	−1.76	0.48	0.0	0.22	0.98	40.3	−0.04	1.04	48.6
IE	NA	NA	NA	2.45	0.67	0.0	1.83	0.63	0.1

For each independent variable we report the mean and SD of the β posterior distribution, and the percentage of the posterior distribution of β estimates (% β) across zero, under the expectation that the distribution of an influential variable is substantially shifted from zero (see Methods). The independent variables in the table are as follows: introduction effort (IE), adult body mass (BM), offspring value index (OV). IE is not included in the analyses of the introduction stage (indicated in the table as ‘NA’).

**Figure 2:**
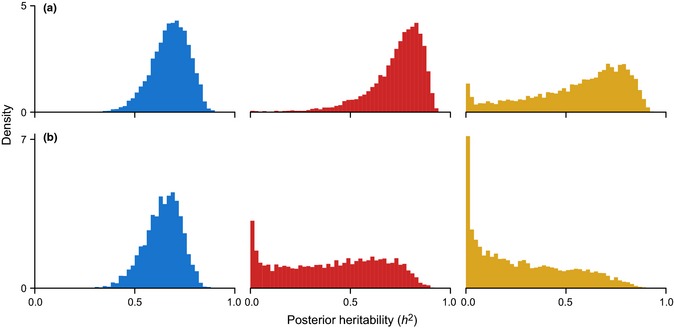
Posterior distribution of heritability (*h* ^2^) at introduction (blue), establishment (red) and spread (yellow). In (a) life history traits, introduction effort and success at each stage along the invasion pathway; in (b) offspring value, adult body mass, introduction effort and success at each stage.

The posterior distribution of heritability shows that heritability is relatively high at each invasion stage in the analyses with all life history traits as well as in those with offspring value (Fig. 2). This indicates an important role of shared ancestry at all stages of invasion and the need to incorporate phylogenetic information when studying biological invasions across species.

## Discussion

Using mammalian historical introductions and up to date phylogenetic comparative approaches, this study quantifies the relative importance of species' life history characteristics to success from introduction to spread, while testing the predictions of two opposing theoretical frameworks on the role of life history traits in sustaining population growth (Pimm 1991; Sæther *et al*. 2004). We thus demonstrate how barriers filter species for success along the invasion pathway in a single taxon at the global scale (Fig. 1c). We show that humans introduce preferentially highly productive species with long reproductive lifespans, characteristics that increase the probability of establishing in novel regions, and higher productivity subsequently sustains the remarkable range expansion typical of invasive mammals. Thus, greater productivity supports population growth as predicted by classic theory (Pimm 1991) and successful alien mammals are ‘hyper‐productive’. Finally, we find that heritability estimates are of intermediate values at all stages of invasion. Our approach thus highlights the importance of accounting for species' shared ancestry with methods that estimate the phylogenetic signal in the data (Supporting Information Section 3).

We find clear evidence that alien mammals are not a random sample from across the mammalian radiation. In the light of recent findings that there may be at least two axes in mammalian life histories, a ‘timing’ and an ‘output’ axis (Bielby *et al*. 2007), barriers at introduction filter mammals that exhibit an unusual combination of traits; high productivity on the output axis but longer reproductive lifespan and more frequent breeding on the timing axis (Fig. 1c). These results are consistent with the suggestion that human preferences for game species or those easy to find, transport and breed, strongly influence the choice of species for introduction (Blackburn & Duncan 2001; Cassey *et al*. 2004b,c; Jeschke & Strayer 2006; Richardson & Pyšek 2012) as many mammalian historical introductions have been intentional (Long 2003).

For the establishment stage, our analysis reveals a more complex picture than either proposed theoretical framework on how founder populations grow and overcome extinction risk suggests (Pimm 1991; Sæther *et al*. 2004). A larger litter size reduces the risk of extinction in alien populations and increases establishment success, as predicted by classic theory (Pimm 1991) but, unexpectedly, with an important albeit weaker contribution of prolonged reproductive lifespan, in line with a more recent theoretical model (Sæther *et al*. 2004) (Fig. 1c). Therefore, population growth from small numbers appears to be supported by life history traits along both the ‘output’ (litter size) and the ‘timing’ axes (reproductive lifespan) in mammals in a way that is not fully predicted by a single theoretical model. Whether this is peculiar to mammals has yet to be established as studies in other vertebrate and plant lineages have considered only a few life history traits. Alternatively, a longer reproductive lifespan might be relevant to population growth only within the context of biological invasions, where a longer reproductive life may allow repeated introductions to the same location, or small neighbouring introduced populations, to merge over short time into a larger population that ultimately enjoys greater establishment success.

To our knowledge, this study is the first to investigate how species traits promote the remarkable range expansion that defines ‘invasive’ species in an animal group at the global scale. We find that, among the established species, successful mammals at the stage of spread produce relatively larger and more frequent litters. Thus, by considering all life history traits in one model, our analysis reveals that alien mammals that colonise vast areas do so through higher fecundity, as predicted by classic theory (Pimm 1991) (Fig. 1c).

The effect of life history traits along the invasion pathway might be even stronger once other factors are also considered, particularly at establishment. For example habitat matching – being introduced in a suitable environment – is likely to have a key influence on success at establishment (Duncan *et al*. 2001; Forsyth *et al*. 2004; Blackburn *et al*. 2011; Richardson & Pyšek 2012; van Wilgen & Richardson 2012) since alien species have not yet had the opportunity to adapt to the new environment. Small founder populations of some species may also experience Allee effects that compromise their success in novel regions (Simberloff 2009). Therefore, habitat matching and Allee effects undermine the ability of a population to increase from low sizes and can potentially reduce any advantage of greater productivity on establishment success. Once established, however, Allee effects and habitat matching might be less important.

By investigating all stages of invasion from introduction to spread, our study demonstrates how barriers at earlier stages affect success at later stages. Given the theoretical models (Pimm 1991; Sæther *et al*. 2004), the same life history traits that underlie population growth at establishment and reduce the risk of extinction when the population is small, should also be important at spread and support the considerable range expansion that characterises biological invasions (Sol 2007; Gurevitch *et al*. 2011; Blackburn *et al*. 2015). We find general support for this suggestion as higher productivity is associated with success at both establishment and spread (Fig. 1c). Bringing all stages together we conclude that, while humans introduce preferentially species that already possess those key traits that enhance species' chances of success in novel environments, the ultimate effect of the barriers at establishment and spread is selecting for more productive species at each stage.

Previous studies show that introduction effort is a strong predictor of establishment success, regardless of how it is measured (Duncan *et al*. 2001; Forsyth & Duncan 2001; Kolar & Lodge 2001; Cassey *et al*. 2004c; Forsyth *et al*. 2004; Marchetti *et al*. 2004; Lockwood *et al*. 2005; Krivánek *et al*. 2006; Sol *et al*. 2008, 2012). Here, we find strong evidence that greater introduction effort increases the probability of establishing in novel regions, and this effect persists at the stage of spread. While small founder populations are under higher extinction risk due to demographic and environmental stochasticity, Allee effects and genetic effects (Simberloff 2009; Richardson & Pyšek 2012; Blackburn *et al*. 2015), once the population has established the first three of these threats should no longer be influential (Lockwood *et al*. 2005; Simberloff 2009; Blackburn *et al*. 2015) while the impact of genetic effects might still persist (Blackburn *et al*. 2015). Thus, our results suggest that greater introduction effort might determine higher genetic variation in the introduced population (Lockwood *et al*. 2005; Simberloff 2009), which in turn should provide greater opportunity to respond to novel environmental conditions through both greater plasticity and evolutionary adaptive potential (Richardson & Pyšek 2012), ultimately leading to long term persistence (Forsman 2015) and range expansion (Blackburn *et al*. 2015).

Biological invasions are often viewed as models to investigate how small populations first escape the risk of extinction and subsequently become widespread (Lockwood *et al*. 2005; Blackburn *et al*. 2015). However, we need to consider the effects of barriers in filtering species across invasion stages when extrapolating results of studies on biological invasions to other systems, such as island colonisation, meta‐communities or species reintroduction. For example, biases should be investigated in the context of island colonisation as barriers to natural dispersal are likely to filter species that reach islands in drastically different ways from how human‐mediated factors select species for introduction to novel locations. However, little is known about whether species traits facilitating arrival on islands lead to biases in other characteristics in the pool of ‘introduced’ island species and, if present, how such biases ultimately affect species' chances of success on islands (Whittaker & Fernandez‐Palacios 2007).

Biological invasions are complex phenomena dependent on the characteristics of the species, the introduction event and the receiving community (van Kleunen *et al*. 2010a; Blackburn *et al*. 2011; Richardson & Pyšek 2012). A holistic understanding of biological invasions therefore requires the integration of community and population level studies at small geographical scales with large scale studies at the whole taxon level. While the former can reveal the peculiarities of each introduction case, the latter are better suited to identify general determinants of invasion success that transcend any regional or local specificity (Hamilton *et al*. 2005; Richardson & Pyšek 2012). Here, we have demonstrated that a comprehensive approach that investigates success in novel regions from introduction to spread at the global scale is necessary to fully unravel the role of species traits in biological invasions. Our results demonstrate the importance of investigating the invasion pathway sequentially and we suggest that barriers at early stages, such as those at introduction documented here, in birds and plants (Blackburn & Duncan 2001; Cassey *et al*. 2004b,c; Jeschke & Strayer 2006; Richardson & Pyšek 2012) and at subsequent stages, should be considered also in studies on island colonisation and species translocation success.

## Authorship statement

IC and CV designed the study; IC, WA, SS and JB developed the protocols for the data collection; SS and WA collected the data; CV and JB analysed the data; IC wrote the ms; all authors provided comments on drafts throughout the manuscript's preparation.

## References

[ele12493-bib-0001] Bielby, J. , Mace, G. , Bininda Emonds, O. , Cardillo, M. , Gittleman, J. , Jones, K. *et al* (2007). The fast‐slow continuum in mammalian life history: an empirical reevaluation. Am. Nat., 169, 748–757. 17479461 10.1086/516847

[ele12493-bib-0002] Blackburn, T.M. & Duncan, R.P. (2001). Establishment patterns of exotic birds are constrained by non‐random patterns in introduction. J. Biogeogr., 28, 927–939.

[ele12493-bib-0003] Blackburn, T.M. , Cassey, P. & Lockwood, J.L. (2009). The role of species traits in the establishment success of exotic birds. Glob. Change Biol., 15, 2852–2860.

[ele12493-bib-0004] Blackburn, T.M. , Pyšek, P. , Bacher, S. , Carlton, J.T. , Duncan, R.P. , Jarošík, V. *et al* (2011). A proposed unified framework for biological invasions. Trends Ecol. Evol., 26, 333–339. 21601306 10.1016/j.tree.2011.03.023

[ele12493-bib-0005] Blackburn, T.M. , Lockwood, J.L. & Cassey, P. (2015). The influence of numbers on invasion success. Mol. Ecol., 24, 1942–1953. 25641210 10.1111/mec.13075

[ele12493-bib-0006] Burns, J.H. , Ashman, T.‐L. , Steets, J.A. , Harmon‐Threatt, A. & Knight, T.M. (2011). A phylogenetically controlled analysis of the roles of reproductive traits in plant invasions. Oecologia, 166, 1009–1017. 21328010 10.1007/s00442-011-1929-9

[ele12493-bib-0007] Carey, J.R. & Judge, D.S. (2000). Longevity Records. Life Spans of Mammals, Birds, Amphibians, Reptiles and Fish. Odense University Press, Odense.

[ele12493-bib-0008] Cassey, P. , Blackburn, T.M. , Jones, K.E. & Lockwood, J.L. (2004a). Mistakes in the analysis of exotic species establishment: source pool designation and correlates of introduction success among parrots (Aves: Psittaciformes) of the world. J. Biogeogr., 31, 277–284.

[ele12493-bib-0009] Cassey, P. , Blackburn, T.M. , Russell, G.J. , Jones, K.E. & Lockwood, J.L. (2004b). Influences on the transport and establishment of exotic bird species: an analysis of the parrots (Psittaciformes) of the world. Glob. Change Biol., 10, 417–426.

[ele12493-bib-0010] Cassey, P. , Blackburn, T.M. , Sol, D. , Duncan, R.P. & Lockwood, J.L. (2004c). Global patterns of introduction effort and establishment success in birds. Proc. R. Soc. B, 271, S405–S408. 10.1098/rsbl.2004.0199 PMC1810115 15801588

[ele12493-bib-0011] DAISIE . (2008). Handbook of Alien Species in Europe. Springer, Dordrecht.

[ele12493-bib-0012] De Magalhaes, J.P. & Costa, J. (2009). A database of vertebrate longevity records and their relation to other life‐history traits. J. Evol. Biol., 22, 1770–1774. 19522730 10.1111/j.1420-9101.2009.01783.x

[ele12493-bib-0013] Duncan, R.P. , Bomford, M. , Forsyth, D.M. & Conibear, L. (2001). High predictability in introduction outcomes and the geographical range size of introduced Australian birds: a role for climate. J. Anim. Ecol., 70, 621–632.

[ele12493-bib-0014] Ernest, S.K.M. (2003). Life history characteristics of placental nonvolant mammals: ecological archives E084‐093. Ecology, 84, 3402.

[ele12493-bib-0015] Forsman, A. (2015). Effects of genotypic and phenotypic variation onestablishment are important for conservation, invasion, and infection biology. PNAS, 111, 302–307. 24367109 10.1073/pnas.1317745111 PMC3890895

[ele12493-bib-0016] Forsyth, D.M. & Duncan, R.P. (2001). Propagule size and the relative success of exotic ungulate and bird introductions to New Zealand. Am. Nat., 157, 583–595. 18707276 10.1086/320626

[ele12493-bib-0017] Forsyth, D.M. , Duncan, R.P. , Bomford, M. & Moore, G. (2004). Climatic suitability, life‐history traits, introduction effort, and the establishment and spread of introduced mammals in Australia. Conserv. Biol., 18, 557–569.

[ele12493-bib-0018] Freckleton, R. , Harvey, P. & Pagel, M. (2002). Phylogenetic analysis and comparative data: a test and review of evidence. Am. Nat., 160, 712–726. 18707460 10.1086/343873

[ele12493-bib-0019] Fritz, S.A. , Bininda‐Emonds, O.R.P. & Purvis, A. (2009). Geographical variation in predictors of mammalian extinction risk: big is bad, but only in the tropics. Ecol. Lett., 12, 538–549. 19392714 10.1111/j.1461-0248.2009.01307.x

[ele12493-bib-0020] Gibson, M.R. , Richardson, D.M. , Marchante, E. , Marchante, H. , Rodger, J.G. , Stone, G.N. *et al* (2011). Reproductive biology of Australian acacias: important mediator of invasiveness? Divers. Distrib., 17, 911–933.

[ele12493-bib-0021] Greene, W.H. (2012). Econometric Analysis. 7 edn. Pearson Education, Harlow.

[ele12493-bib-0022] Gurevitch, J. , Fox, G.A. , Wardle, G.M. , Inderjit & Taub, D. (2011). Emergent insights from the synthesis of conceptual frameworks for biological invasions. Ecol. Lett., 14, 407–418. 21513009 10.1111/j.1461-0248.2011.01594.x

[ele12493-bib-0023] Hadfield, J.D. (2010). MCMC methods for multi‐response generalized linear mixed models: the MCMCglmm R package. J. Stat. Softw., 33, 1–22.20808728

[ele12493-bib-0024] Hamilton, M.A. , Murray, B.R. , Cadotte, M.W. , Hose, G.C. , Baker, A.C. , Harris, C.J. *et al* (2005). Life‐history correlates of plant invasiveness at regional and continental scales. Ecol. Lett., 8, 1066–1074.

[ele12493-bib-0025] Hui, C. , Richardson, D.M. , Robertson, M.P. , Wilson, J.R.U. & Yates, C.J. (2011). Macroecology meets invasion ecology: linking the native distributions of Australian acacias to invasiveness. Divers. Distrib., 17, 872–883.

[ele12493-bib-0026] IUCN (Ed.). (2013). Global Invasive Species Database (GISD). URL http://www.issg.org/database/welcome/. Last accessed 15th of July 2014.

[ele12493-bib-0027] Jeschke, J.M. & Strayer, D.L. (2006). Determinants of vertebrate invasion success in Europe and North America. Glob. Change Biol., 12, 1608–1619.

[ele12493-bib-0028] Jones, K.E. , Bielby, J. , Cardillo, M. , Fritz, S.A. , O'Dell, J. , Orme, C.D.L. *et al* (2009). PanTHERIA: a species‐level database of life history, ecology, and geography of extant and recently extinct mammals. Ecology, 90, 2648.

[ele12493-bib-0029] van Kleunen, M. , Dawson, W. , Schlaepfer, D. , Jeschke, J.M. & Fischer, M. (2010a). Are invaders different? A conceptual framework of comparative approaches for assessing determinants of invasiveness. Ecol. Lett., 13, 947–958. 20576028 10.1111/j.1461-0248.2010.01503.x

[ele12493-bib-0030] van Kleunen, M. , Weber, E. & Fischer, M. (2010b). A meta‐analysis of trait differences between invasive and non‐invasive plant species. Ecol. Lett., 13, 235–245. 20002494 10.1111/j.1461-0248.2009.01418.x

[ele12493-bib-0031] Kolar, C.S. & Lodge, D.M. (2001). Progress in invasion biology: predicting invaders. Trends Ecol. Evol., 16, 199–204. 11245943 10.1016/s0169-5347(01)02101-2

[ele12493-bib-0032] Krivánek, M. , Pyšek, P. & Jarošík, V. (2006). Planting history and propagule pressure as predictors of invasion by woody species in a temperate region. Conserv. Biol., 20, 1487–1498. 17002766 10.1111/j.1523-1739.2006.00477.x

[ele12493-bib-0033] Lockwood, J.L. , Cassey, P. & Blackburn, T. (2005). The role of propagule pressure in explaining species invasions. Trends Ecol. Evol., 20, 223–228. 16701373 10.1016/j.tree.2005.02.004

[ele12493-bib-0034] Long, J.S. (1997). Regression Models for Categorical and Limited Dependent Variables. SAGE Publications Inc, London.

[ele12493-bib-0035] Long, J.L. (2003). Introduced Mammals of the World: Their History. CABI Publishing, Distribution and Influence.

[ele12493-bib-0036] Marchetti, M.P. , Moyle, P.B. & Levine, R. (2004). Invasive species profiling? Exploring the characteristics of non‐native fishes across invasion stages in California. Freshwater Biol., 49, 646–661.

[ele12493-bib-0037] Pimm, S. (1991). The Balance of Nature?. University of Chicago Press, Chicago.

[ele12493-bib-0038] Richardson, D.M. & Pyšek, P. (2012). Naturalization of introduced plants: ecological drivers of biogeographical patterns. New Phytol., 196, 383–396. 22943470 10.1111/j.1469-8137.2012.04292.x

[ele12493-bib-0039] Richardson, D.M. & Rejmanek, M. (2004). Conifers as invasive aliens: a global survey and predictive framework. Divers. Distrib., 10, 321–331.

[ele12493-bib-0040] Sæther, B.E. , Engen, S. , Pape Møller, A. , Weimerskirch, H. , Visser, M.E. , Fiedler, W. *et al* (2004). Life history variation predicts the effects of demographic stochasticity on avian population dynamics. Am. Nat., 164, 793–802. 10.1086/425371 29641930

[ele12493-bib-0041] Seger, J. & Brockmann, H.J. (1987). What is bet‐hedging? In Oxford Surverys in Evolutionary Biology (ed Harvey P.H.). Oxford University Press, Oxford, pp. 182–211.

[ele12493-bib-0042] Simberloff, D. (2009). The role of propagule pressure in biological invasions. Annu. Rev. Ecol. Evol. Syst., 40, 81–102.

[ele12493-bib-0043] Sol, D. (2007). Do successful invaders exist? pre‐adaptations to novel environments in terrestrial vertebrates In: Biological Invasions. Ecological Studies (ed. Nentwig W.). Springer‐Verlag, Berlin, pp. 127–141.

[ele12493-bib-0044] Sol, D. , Bacher, S. , Reader, S.M. & Lefebvre, L. (2008). Brain size predicts the success of mammal species introduced into novel environments. Am. Nat., 172, S63–S71. 18554145 10.1086/588304

[ele12493-bib-0045] Sol, D. , Maspons, J. , Vall‐Llosera, M. , Bartomeus, I. , García‐Peña, G.E. , Piñol, J. *et al* (2012). Unraveling the life history of successful invaders. Science, 337, 580–583. 22859488 10.1126/science.1221523

[ele12493-bib-0046] de Villemereuil, P. , Gimenez, O. & Doligez, B. (2012). Comparing parent‐offspring regression with frequentist and Bayesian animal models to estimate heritability in wild populations: a simulation study for Gaussian and binary traits. Methods Ecol. Evol., 4, 260–275.

[ele12493-bib-0047] Whittaker, R.J. & Fernandez‐Palacios, J.M. (2007). Island Biogeography, 2nd edn Oxford University Press, Oxford.

[ele12493-bib-0048] van Wilgen, N.J. & Richardson, D.M. (2012). The roles of climate, phylogenetic relatedness, introduction effort, and reproductive traits in the establishment of non‐native reptiles and amphibians. Conserv. Biol., 26, 267–277. 22236256 10.1111/j.1523-1739.2011.01804.x

