# Supplementary material for: The role of life history traits in mammalian invasion success
Source: Ecol Lett. 2015 Aug 21;18(10):1099–107. doi: 10.1111/ele.12493 (PMC4989474; doi:10.1111/ele.12493)
Supplement: Supplementary file 1 [file ELE-18-1099-s001.docx]

SUPPLEMENTARY INFORMATION

1 – METHODS 2

1.1 – Data collection 2

1.1.1 – Protocol for the classification and status of alien species 2

1.1.2 – Classification of alien mammals at the species level 6

1.1.3 – Introduction effort 9

1.1.4 – Offspring value index (OV) 11

1.2 – Statistical analysis 12

1.2.1 – Estimating the effect sizes for the life history variables: average partial effects (APEs) 12

1.2.2 – Robustness of the results to multicollinearity 13

1.2.3 – Associations between offspring value index and life history traits 14

1.2.4 – Potential sampling biases 15

2 – SUPPLEMENTARY RESULTS 15

2.1 – Multicollinearity: variance inflation factors (VIF) and reduced models 15

2.2 – Offspring value and life history traits 21

2.3 – Potential sampling biases 22

2.4 – Alternative transformations for introduction effort 24

3 – SUPPLEMENTARY DISCUSSION 28

4 – SUPPLEMENTARY REFERENCES 31

4.1 – Supplementary references 31

4.2 – Supplementary data references 35

1 – METHODS

1.1 – Data collection

1.1.1 – Protocol for the classification and status of alien species

Following the ecological frameworks of invasion (Blackburn *et al.* 2011), we define the status of a species with respect to the 3 stages of invasion as follows:

Introduction: a species is considered ‘introduced’ if released by humans, intentionally or accidentally, in the wild outside its native range at least once. Species described as ‘exotic’, ‘alien’, ‘invasive’, ‘introduced’, ‘naturalized’, ‘released’, ‘non-native’ or similar are classified as introduced only if they conform to this definition. Introductions to national parks, natural reserves and game parks are included in the dataset if the populations are free ranging and not provisioned. Conversely, introductions into confined environments such as ‘ranches’, safari parks, captive enclosures, or similar, where animals are food provisioned, are discarded unless animals are subsequently released into the wild or escape from confines. We also ignore any cases where it cannot be confidently determined that an introduction location is outside of the native range, and any unconfirmed introduction (e.g. defined as ‘possible’ or similar). Moreover, we do not classify as ‘alien’ those populations that have been reintroduced in areas where the species was formerly present (i.e. within its historical native range). Likewise, records of ‘alien’ populations that originate from expansion of their range into new areas by their own means (e.g. by natural dispersal as a consequence of climatic changes) are not considered as evidence of introduction. Of a total of 518 species in the main analysis for which all life history traits are available, 172 are classified as introduced following these criteria.

Establishment: within the subset of the introduced mammals, a species is considered successfully established if there is evidence of at least one introduced population that is self-sustaining, i.e. a population that successfully reproduces and persists in the wild in a non-native locality for a time interval equal to or greater than the maximum lifespan of the species, without any form of supplementary care. This temporal interval ensures that an introduced population has had sufficient time to establish. This protocol is therefore applicable also to cases where an alien population is recorded as ‘still present’ or ‘established’ without any specific mention of successful reproduction. We check the time elapsed from the reported introduction date against the species’ longest maximum lifespan as recorded in PanTHERIA (Jones *et al.* 2009), AnAge (De Magalhaes & Costa 2009), Ernest (2003) and Carey and Judge (2000), or alternative sources if unavailable from these references (full data reference list in section 4.2). Records of persistence for an introduced population are discarded when time since introduction is less than this time interval. When the date of introduction is not available, we assess the time interval between the date of publication of the earliest source reporting the presence of the alien population and its latest recorded presence, and use this as a surrogate for evaluating establishment success of the alien population in the same fashion as described above. Specifically, if the time interval between publications recording a population as ‘established’ or ‘present’ is equal to or greater than the maximum lifespan of the species, we consider this as evidence of successful establishment. For multiple introductions into the same locality, we calculate the time interval against which to assess establishment success from the date of the last introduction to the date of the latest recorded presence. We also consider a population successfully established if there is clear and unambiguous evidence that a species was introduced centuries ago by humans and is still present. Eradications by humans of alien populations are not counted as failures at this stage if the species has initially established according to the criteria here defined. We discard remaining cases where no introduction date is available and assessment through date of publication of multiple sources is not possible. Introductions with unknown or uncertain outcomes are also discarded. Finally, we discard cases of alien populations that originate from continuous releases from fur farms if it is not possible to determine whether a species is truly established and reproducing in the wild, or whether its presence results exclusively from constant releases. We consider a species as not established if (i) there is no evidence of at least one introduced population that has established successfully following the criteria here outlined, and additionally (ii) there is evidence of at least one introduced population that has failed to establish. Of 172 introduced species with all life history traits, 121 are classified as successful and 28 as unsuccessful at establishment stage following these criteria; for 23 introduced species the outcome of their introductions is uncertain and are therefore excluded from the analysis at establishment and spread stages.

Spread: within the subset of successfully established mammals, a species is classified as successful at spread if at least one established population exhibits remarkable range expansion from the initial location of introduction (van Kleunen *et al.* 2010a; 2010b; Blackburn *et al.* 2011). Words such as ‘widespread’, ‘common’, ‘expanding’, ‘flourishing’, ‘invasive’, ‘pest’, or similar are considered evidence of successful spread if in conjunction with detailed description of range and/or population expansion. Populations that are described, as ‘restricted’, ‘not expanding beyond the location of introduction’ or similar, or populations with very limited range expansion, are classified as unsuccessful at this stage. Cases of range expansion across small islands (<50,000km^2^) are discarded as we consider the available area too small to adequately assess success at spread as defined above; however, we consider lack of spread within small islands as evidence of failure to spread. Species classified as unsuccessful at spread are therefore those for which none of the established populations, with sufficient information and not introduced on small islands, exhibits remarkable range expansion. For cases where wording associated with success at spread, as described above, is only mentioned in brief descriptions without any supporting evidence in the original source, we look for further evidence of substantial range expansion from additional sources (section 4.2) and use any qualitative information in the same fashion as described above. When recent maps of the extent of invaded range are available we use these to resolve, confirm, or update original records on whether range expansion is remarkable given the date of introduction. Typically this leads to confirming the qualitative description or provides information where none is available in the original source. All ambiguous cases are thoroughly investigated and classified independently by at least two authors. All these contentious cases are then discussed and, if the information is still insufficient to make a decision or not available, we discard the original record from the dataset as no judgement can be made with confidence. For a few species where no decision can be reached on the success at spread for any of its alien populations, the species are excluded from the dataset of species at stage of spread (but retained for the analyses at introduction and establishment as no ambiguity is present at those stages). Of the 121 established species with all life history traits, 47 are classified as successful at spread and 27 as unsuccessful, while 47 species are excluded from the analysis at this stage due to lack of sufficient information (i.e. of all established populations across all species that are discarded*:* approximately 65% are on small islands; less than 1% are excluded due to post-establishment eradication; about 8% have insufficient data and for about 25% no additional information is available).

Using this protocol, we extract data on invasion success from three main sources (Long 2003; DAISIE 2008; IUCN 2013) complemented by additional sources (full data reference list in section 4.2). To ensure that our classification at both the population and the species level is robust and objective, all records and information for 10% randomly selected species and all the critical records that determine the outcome at spread of all the successfully established species were double checked by at least two authors. Moreover, at least two authors double-checked all information regarding all ambiguous cases in the original source for all stages of invasion, and the classification resulting after additional checks and information are found (if available); for cases where a decision cannot be made at a stage, ambiguous records are discarded. Where necessary, we update species’ names to match the taxonomy of Wilson & Reeder (Wilson & Reeder 2005).

1.1.2 – Classification of alien mammals at the species level

Here we are interested in investigating whether a species has the potential to invade and exhibits preadaptations that promote its success in novel environments, rather than in explaining which characteristics of the introduction sites or receiving communities explain the success of alien populations. Thus, although success at a stage is a population level event, we consider one successful event (i.e. one alien population) as sufficient evidence to demonstrate that a species, as a whole, has the potential to succeed at that stage. In support of this approach, studies on invasive species at smaller geographical scales demonstrate that ‘success elsewhere’ is a very strong predictor of success of newly introduced alien populations of the same species in a novel region. For example, while controlling for introduction effort, a history of successful invasion elsewhere in the world is a strong predictor of establishment success in mammals and birds introduced to Australia, reptiles and amphibians introduced to California, Florida and Britain, and alien fish in California (Duncan *et al.* 2001; Forsyth *et al.* 2004; Marchetti *et al.* 2004; Bomford *et al.* 2009), and alien plants (Kolar & Lodge 2001). Furthermore, Forsyth *et al.* (2001) show that, after controlling for introduction effort, within species variation in outcomes across locations for ungulate and bird species introduced to New Zealand is explained by unsuitable habitats at the introduction location. Similar results have been found in plants (Richardson & Pyšek 2012). Finally, in taxonomically based studies using success/failure at the event (i.e. population) level ranked within species, species identity is one of the strongest predictors of success (e.g. Tingley *et al.* 2011; Rago *et al.* 2012; Sol *et al.* 2012). Altogether this evidence strongly suggests that a species classification for success is justified and appropriate when investigating species’ potential to invade, as successful invaders in one location are more likely to succeed than to fail in other locations, and failures at one location of successful invaders elsewhere are most likely explained by introductions to unsuitability of habitats and low introduction effort.

Several studies on invasive species model success as a location level event nested within species, but mostly either ignore (i.e. analyses without phylogeny), overestimate (e.g. with independent contrasts) or underestimate (e.g. using taxonomy as a proxy of phylogeny) species’ similarity due to shared ancestry. Here we use novel phylogenetic comparative approaches for binary dependent variables to quantify the phylogenetic signal in the data (Hadfield 2010). In phylogenetic comparative analyses across species, the species is the unit of analysis and individual events of introduction for the same species are modeled as intraspecific variation, treated as a random effect (Hadfield 2010). However, simulations and empirical analyses demonstrate that within species variation in phylogenetic comparative analyses only slightly improves parameter estimates and the estimate of the phylogenetic signal, but crucially it does not alter either the direction or the significance of model parameters for the fixed effects (Ives *et al.* 2007; Revell & Graham Reynolds 2012). Thus, including event level outcomes at a stage for every species with multiple introductions in our analyses would not alter our conclusions, particularly when also considering that life history traits would not vary across locations for a species. Conversely, such an approach would heavily reduce sample sizes of species by up to 40% due to missing data on the outcomes of individual introductions (see below) and consequently the power of the analyses.

Specifically, including event level outcomes for a species to be modeled as within species variation would require that a different measure of introduction effort is used, as number of locations would no longer be applicable (since locations would be modeled as within species variation). Therefore, a location level approach would require that introduction effort is estimated as number of introduced individuals for each alien population. There is however no information or only qualitative information on number of introduced individuals for approximately 75% of the recorded mammalian introductions in our dataset. This will have two major consequences, namely: (i) the extent of within species variation would be much reduced with approximately 40% of species having data for only one alien population, and (ii) the sample size of species would be reduced by about 40% at establishment and 30% at spread, compromising greatly the power of analyses aiming to test all the life history traits and introduction effort in a single model.

Conversely, our approach to classify a species as successful at a stage if at least one of its alien populations has succeeded at that stage allows us to: (i) use all available information about the status of alien populations for a species, regardless of whether information on the number of introduced individuals is available (see also section 1.1.3 for how we control for introduction effort); (ii) have a large and sufficient sample of species to test the predictions of the two opposing theoretical models on population growth (Pimm 1991; Sæther *et al.* 2004) at both establishment and spread while quantifying the relative effect of each life history traits on success (and so account for the correlated evolution between life history traits; see section 1.2.2); and (iii) account for the phylogenetic signal in the data as estimated by the model (Hadfield 2010). Our approach is supported by independent evidence that successful species in one location are more likely to succeed in other locations, that failed introductions of successful species are mostly caused by habitat unsuitability, and that species identity is a strong predictor of success. Our approach is also justified by independent studies showing that parameter estimates and the significance of the fixed effects in phylogenetic comparative analyses are qualitatively unaltered when within species variation is included.

1.1.3 – Introduction effort

It is well established that higher introduction effort (also called ‘introduction pressure’ or ‘propagule pressure’) strongly increases the chances of successful establishment (Cassey *et al.* 2004; Lockwood *et al.* 2005; Simberloff 2009; Sol *et al.* 2012). Ignoring this factor can lead to erroneous conclusions in comparative analyses on the determinants of invasion success across species (Cassey *et al.* 2004). The magnitude of introduction effort is dependent on the number of introduced individuals and introduction events to the same locality, and the number of unique introduction locations (Lockwood *et al.* 2005; Simberloff 2009; Blackburn *et al.* 2011). These factors are positively associated with one another, and they all increase the probability of a species to successfully establish; species that are introduced in larger numbers, more frequently, and in more locations have higher chances to establish in non-native regions (Duncan *et al.* 2001; Forsyth & Duncan 2001; Cassey *et al.* 2004; Forsyth *et al.* 2004; Lockwood *et al.* 2005; van Wilgen & Richardson 2012). Moreover, studies on the establishment success of alien mammals (Forsyth *et al.* 2004) and birds (Duncan *et al.* 2001) in Australia show that the number of unique locations of introduction is as strong (or better) a determinant of success as the other two factors.

We extract data on the number of unique introduction locations from the sources reporting the status of alien mammals (Long 2003; DAISIE 2008; IUCN 2013) (see also section 4.2). We count repeated introductions to the same unique locality as one introduction location, but multiple localities in the same region or country as unique introduction locations. When detailed information is not available and a species is reported as being introduced to a region, we adopt a conservative approach and count the whole region as one introduction location. For example, Guinea pigs (*Cavia porcellus*) have been introduced to New Zealand but no further details are reported (Long 2003); we thus count this as one introduction location. Conversely, axis deer (*Cervus axis*) are introduced to multiple named locations and islands of New Zealand; we count these as separate introduction locations (Long 2003).

For approximately 25% of the recorded introductions across all species in our dataset, the number of introduced individuals is also known. For each species we thus record the total number of introduced individuals across all introduction locations (Sum individuals), and the largest number of introduced individuals in a single location (Largest introduction). As in previous studies in mammals and birds at small geographical scale (Duncan *et al.* 2001; Forsyth & Duncan 2001; Cassey *et al.* 2004; Forsyth *et al.* 2004), we find that the number of introduction locations (N locations) is strongly and positively associated with the number of introduced individuals across species at the global scale, regardless of how the number of introduced individuals is quantified (Pearson’s correlation for: N locations vs Sum individuals r=0.78, T_88_=11.71, p<0.001; N locations vs Largest introduction: r=0.69, T_88_=8.96, p<0.001). These results, together with the fact that the number of introduction locations is a strong predictor of success regardless of how we analyse it (see Section 2.4), demonstrate that the number of introduction locations is a very good estimate of the magnitude of introduction effort across species. Therefore, following previous studies (Duncan *et al.* 2001; Forsyth *et al.* 2004; Krivánek *et al.* 2006; van Wilgen & Richardson 2012), we use the number of unique locations of introduction as an estimate of introduction effort to maximize sample sizes, since other measures of introduction effort are available for fewer mammals in our dataset.

1.1.4 – Offspring value index (OV)

We calculate an ‘offspring value’ (OV) index as a measure of current versus future reproductive effort following previous studies (Bókony *et al.* 2009; Sol *et al.* 2012) as:

$$\mathbf{Equation 1}: OV=\frac{1}{(Litters per year)*(Reproductive Lifespan)}$$

Recent theoretical models of how small populations overcome extinction risks in variable environments propose that longer lived species are more likely to succeed and establish than shorter lived, highly fecund species (Sæther *et al.* 2004; Jeppsson & Forslund 2012). Relative to short lived species, long lived organisms can adopt more flexible strategies, such as bet-hedging, when facing temporally and/or spatially heterogeneous or stochastic environments. Specifically, they can postpone reproduction in unfavourable years and invest into a greater reproductive output in more favourable years. In the context of these models, a low OV is predicted to be associated with higher probability of establishment of alien populations and so the association between success and OV should be negative (Sol *et al.* 2012). A recent study in birds finds support for this model and reveals that a low OV can be achieved through a variable combination of clutch size and lifespan (Sol *et al.* 2012). Specifically, successfully established alien birds with low OV produce either many clutches per a year over short reproductive lifespan or a single annual clutch over a long reproductive life. This study also shows that OV in birds is unrelated to other life history traits, suggesting that OV captures an independent dimension of the species’ life history strategy (Sol *et al.* 2012).

Here we assess the relationship between OV and life history traits (section 2.2) to investigate whether OV represents an independent life history axis in mammals, as suggested for birds (Sol *et al.* 2012).

1.2 – Statistical analysis

1.2.1 – Estimating the effect sizes for the life history variables: average partial effects (APEs)

We use average partial effects (APEs) as a measure of the effect size of the continuous life history traits (Long 1997; Greene 2012) that have been identified as influential in the main analysis. APEs are estimates of the probability of change in the response variable (from 0 to 1 and *vice versa*) for a unit change in a given independent variable, averaged across all observed values of all independent variables in the model (Long 1997; Greene 2012). For each independent variable we derive the posterior distributions of its APE from the posterior distributions of its *β* estimates, i.e. we estimate the APE for every iteration of the posterior *β* values, using the following equation (Greene 2012):

$$\mathbf{Equation 2}: APE of x_{c}= \sum_{i=1}^{n} \frac{f(x_{i}^{'}\hat{\beta})\hat{\beta_{c}}}{n}$$

Where $f$ is the normal density function (*ϕ*) for a probit model, $\hat{\beta_{c}}$ is the parameter estimate of a continuous independent variable $x_{c}$, ($x_{i}^{'}\hat{\beta})$ are the fitted model values for each independent variable, and $n$ is the number of observations. At a single iteration, Equation 2 quantifies the contribution of the parameter estimate ($\hat{\beta_{c}}$) for a continuous independent variable ($x_{c}$) to the fitted model values ($x_{i}^{'}\hat{\beta})$at every observed value of all other independent variables in the model, and therefore estimates the average partial effect of $x_{c}$ at that iteration (Mood 2010). In a Bayesian framework, APEs are calculated at each iteration of the posterior distribution of estimates, resulting in a posterior distribution of APEs for each independent variable.

1.2.2 – Robustness of the results to multicollinearity

Some life history traits covary in mammals along at least two life history axes (Bielby *et al.* 2007). Therefore we assess whether possible multicollinearity between all the predictors (i.e. life history traits and introduction effort where appropriate) in our models has any effect on our conclusions.

Variance inflation factors (VIF) are commonly used to quantify how much the variance of a given parameter is increased in the presence of multicollinearity (Quinn & Keough 2007). We calculate VIFs for all life history traits from non-phylogenetic GLS regressions. This is a conservative approach since it is well established that the strength of the associations between life history traits is weakened once phylogeny is accounted for, and therefore VIFs are likely to be higher in a non-phylogenetic analysis than in a phylogenetic comparative analysis accounting for species’ shared ancestry. VIFs are calculated as:

$${\mathbf{Equation 5:} VIF}_{k}=\frac{1}{(1- {R_{k}}^{2})}$$

where R_k_^2^ is the R^2^ value obtained by regressing the *k^th^* predictor on the remaining predictors. VIF values greater than 5 are considered evidence of possibly problematic collinearity and greater than 10 of strong collinearity (Quinn & Keough 2007).

When we find evidence of possibly problematic multicollinearity in a model, we first remove the life history variable with the highest VIF value from the model. We then recalculate VIFs for the new reduced model, and if any remaining variable has a VIF greater than 5, repeat the procedure until all remaining variables have VIFs lower than 5. We call these models ‘reduced models’ as they contain only the subset of life history traits with VIFs lower than 5. We then repeat the analyses of probability of success at each stage of invasion in MCMCglmm (with the same specifications as the main analysis, as described in the main text) with these ‘reduced models’ and assess the relative contribution of each independent variable as described in sections 1.2.1.

In section 2.1 we report the VIFs for all life history variables and introduction effort in the full and reduced models, and the results of the reduced models (mean and SD of the posterior distribution of *β* estimates for each remaining independent variable in the reduced models and percentage of their posterior distribution crossing zero; for details on the methods see main text and 1.2.1).

1.2.3 – Associations between offspring value index and life history traits

We assess whether the offspring value index (OV, see 1.1.3) exhibits any association with each life history trait individually using phylogenetic generalized linear models in MCMCglmm (Hadfield & Nakagawa 2010). We use normally distributed priors with a mean of 0 and a large variance around the mean (10^8^) for the life history traits treated as fixed effects (Hadfield & Nakagawa 2010; Hadfield 2012), and a proper Cauchy prior with wide scaling variance (10^8^) for the phylogeny treated as a random effect (Hadfield & Nakagawa 2010; Hadfield 2012). The MCMC chains are run as described in the main text. This analysis is repeated for each stage of invasion. Here we report the mean and SD of the posterior distribution of *β* estimates for the slope of the pairwise association between OV and each life history traits, and the percentage of the *β* posterior distribution crossing over 0 (main text).

1.2.4 – Potential sampling biases

The dataset for the main analysis includes mammals for which data on *all* life history traits are available (n=518, introduction stage; main text and section 1.1.1). We assess potential sampling biases with regard to life history traits by checking whether our total sample of species for the main analysis is a random sample from across the mammalian phylogeny. To do so we test if the mean values of each life history trait differ between mammals in the dataset for the main analysis (with all life history data; ‘Included’ sample, n=518) and mammals that lack data for at least one life history trait (‘Excluded’ sample). We thus code each mammalian species as either ‘included’ or ‘excluded’ in the dataset for the main analysis and perform a phylogenetic t-test in maximum likelihood within PGLS in BayesTraits (Pagel *et al.* 2004), with the life history trait of interest as the dependent variable, and the classification of the species as ‘Included’ in the main analysis (coded as 1) or not (coded as 0, i.e. ‘Excluded-all’ sample) as independent binary variable.

2 – SUPPLEMENTARY RESULTS

2.1 – Multicollinearity: variance inflation factors (VIF) and reduced models

Some life history traits in the full model (with all the independent variables) have VIFs greater than 5 (Table S1). According to the protocol described in section 1.2.2, we exclude the following variables from the models: neonatal body mass and age at first birth for introduction, neonatal body mass for establishment, and neonatal and adult body mass for spread. The VIF values of all the independent variables retained in these reduced models are below 5, indicating that multicollinearity is no longer potentially problematic (Table S1).

Next we re-run the analyses on the probability of success at each invasion stage with only the independent variables retained in the reduced models, with the same model specification as the full models (see main text and section 1.2.2). These produce qualitatively similar results to those presented in the main text (full models; Tables S2). Specifically, we find again strong effects of larger litter size, more litters per year and longer reproductive lifespan on the probability of being introduced in non-native environments (Tables S2a). Thus, the APEs (section 1.2.1) indicate that the relative probability of being introduced increases by approximately 3.9% for every additional offspring produced in a litter, 2.0% for every additional litter produced per year, and 3.9% for every additional day of reproductive lifespan (Table S2a). At establishment, litter size, reproductive lifespan and introduction effort are influential on the probability of success (Table S2b), such that every additional offspring produced increases the relative probability of establishing by 3.8% and a day longer reproductive lifespan by 1.8% (Table S2b). At spread, larger and more frequent litters, and higher introduction effort increase the probability of success at this stage as in the main analysis, but we also find a weaker effect of older weaning age (Tables S2c). Consistent with the main analysis (main text, Figure 1b), the relative probability of successfully spreading into non-native regions increases of 3.8% and 5.2% for every additional litter produced in a year and every additional offspring in a litter respectively, and 3.0% for every additional day of lactation (Table S2c). Altogether this analysis confirms our main result that introduced species produce larger and more frequent litters over a longer reproductive lifespan; greater introduction effort, larger litters and longer reproductive lifespan increase the probability of success at establishment, while success at spread is associated with higher productivity, greater introduction effort and possibly with marginally longer postnatal maternal investment (Tables S2). The heritability values are qualitatively similar between the full (main text, Figure 2a) and reduced model (Figure S1).

**Table S1**. Variance inflation factors (VIF) of the independent variables in the full models and the reduced models at each invasion stage (introduction, establishment and spread). Values of VIF greater than 5 are considered potentially problematic (see section 1.2.5 for details). The independent variables in the table are reported as follows: adult body mass (BM), gestation time (GT), weaning age (WA), neonatal body mass (NBM), litter size (LS), litters per year (LY), age at first birth (AFB), reproductive lifespan (RL), introduction effort (IE). For the reduced models ‘NA’ indicate that a given independent variable has been excluded from the models due to its high VIF value in the full model.

| Invasion stage | Introduction |  | Establishment |  | Spread |  |
| --- | --- | --- | --- | --- | --- | --- |
| VIF for: | Full model | Reduced model | Full model | Reduced model | Full model | Reduced model |
| BM | 8.45 | 3.29 | 6.99 | 3.88 | 9.19 | NA |
| GT | 9.38 | 4.34 | 11.88 | 3.07 | 15.41 | 3.00 |
| WA | 3.27 | 2.82 | 3.78 | 3.05 | 5.14 | 3.60 |
| NBM | 12.99 | NA | 14.79 | NA | 20.86 | NA |
| LS | 2.88 | 2.78 | 3.42 | 3.13 | 3.34 | 3.32 |
| LY | 3.32 | 2.62 | 2.87 | 2.76 | 2.43 | 2.30 |
| AFB | 5.08 | NA | 3.42 | 3.35 | 2.63 | 2.43 |
| RL | 3.52 | 3.28 | 2.84 | 2.82 | 2.74 | 2.62 |
| IE | NA | NA | 1.22 | 1.22 | 1.33 | 1.14 |

**Table S2.** Comparison of the results between the full model (with all life history traits, as presented in the main text; left columns in this table) and the reduced models (where some life history variables have been excluded after controlling for multicollinearity, right columns, see Table S1). For each independent variable we report the mean and SD of the *β* posterior distribution, and the percentage of *β* posterior distribution (% *β*) beyond zero (see main text for details). Stages of invasion: in (a) introduction, in (b) establishment, and in (c) spread. The independent variables in the table are reported as follows: introduction effort (IE), adult body mass (BM), gestation time (GT), weaning age (WA), neonatal body mass (NBM), litter size (LS), litters per year (LY), age at first birth (AFB), reproductive lifespan (RL). For the reduced models ‘NA’ indicates that a given independent variable was not included.

| (a) Introduction | Full model | | | Reduced model | | |
| --- | --- | --- | --- | --- | --- | --- |
| Statistics | Mean *β* | SD *β* | % *β* | Mean *β* | SD *β* | % *β* |
| BM | 0.38 | 0.41 | 17.1 | 0.12 | 0.22 | 28.1 |
| GT | -0.77 | 1.02 | 22.1 | -1.06 | 0.89 | 11.3 |
| WA | 0.03 | 0.54 | 46.7 | 0.12 | 0.52 | 40.7 |
| NBM | -0.38 | 0.49 | 20.4 | NA | NA | NA |
| LS | 2.91 | 0.91 | 0.0 | 3.01 | 0.83 | 0.0 |
| LY | 1.66 | 0.75 | 1.0 | 1.52 | 0.69 | 1.4 |
| AFB | 0.25 | 0.58 | 33.6 | NA | NA | NA |
| RL | 2.99 | 0.74 | 0.0 | 3.00 | 0.71 | 0.0 |

| (b) Establishment | Full model | | | Reduced model | | |
| --- | --- | --- | --- | --- | --- | --- |
| Statistics | Mean *β* | SD *β* | % *β* | Mean *β* | SD *β* | % *β* |
| BM | -0.30 | 0.85 | 36.0 | -0.18 | 0.57 | 38.3 |
| GT | -2.20 | 2.41 | 17.5 | -1.94 | 1.81 | 12.8 |
| WA | 0.90 | 1.41 | 26.0 | 0.82 | 1.31 | 25.8 |
| NBM | 0.15 | 0.97 | 42.9 | NA | NA | NA |
| LS | 5.51 | 2.70 | 0.9 | 5.23 | 2.61 | 0.8 |
| LY | 1.44 | 2.21 | 25.9 | 1.41 | 2.13 | 25.4 |
| AFB | 0.61 | 1.62 | 34.9 | 0.59 | 1.58 | 34.8 |
| RL | 2.49 | 1.92 | 8.9 | 2.39 | 1.85 | 8.9 |
| IE | 2.70 | 0.85 | 0.0 | 2.61 | 0.82 | 0.0 |

| (c) Spread | Full model | | | Reduced model | | |
| --- | --- | --- | --- | --- | --- | --- |
| Statistics | Mean *β* | SD *β* | % *β* | Mean *β* | SD *β* | % *β* |
| BM | 0.28 | 0.85 | 36.3 | NA | NA | NA |
| GT | -0.54 | 3.28 | 43.1 | -0.58 | 1.83 | 38.1 |
| WA | 1.96 | 1.83 | 13.8 | 2.10 | 1.63 | 9.1 |
| NBM | -0.23 | 1.11 | 42.5 | NA | NA | NA |
| LS | 3.93 | 2.30 | 3.7 | 3.70 | 2.07 | 2.6 |
| LY | 3.15 | 2.39 | 8.2 | 2.79 | 2.16 | 8.3 |
| AFB | 1.55 | 1.53 | 15.4 | 1.36 | 1.35 | 15.4 |
| RL | 0.31 | 1.84 | 43.1 | 0.33 | 1.59 | 41.4 |
| IE | 1.77 | 0.82 | 1.0 | 1.72 | 0.74 | 0.6 |

**Figure S1.** Posterior distribution of heritability (*h^2^*) at introduction (blue), establishment (red) and spread (yellow) for the reduced model in which multicollinearity is resolved.

2.2 – Offspring value and life history traits

The offspring value index (OV) has been proposed and used as an estimate of current versus future reproductive investment in previous studies (Bókony *et al.* 2009; Sol *et al.* 2012) (see main text and section 1.1.3). In birds this index is unrelated to life history traits, suggesting that it represents another life history axis in this lineage (Sol *et al.* 2012). In mammals, however, we find that low OV (indicating that reproductive investment is divided in more reproductive attempts during the reproductive lifespan of a species) is associated with larger litters, fewer litters per year, faster life history traits and smaller body mass, at all stages of invasion (Table S3). These findings, together with the results showing stronger associations between life history traits and success at each invasion stage (main text) relative to those found with OV (main text), suggest that the importance of OV for success at introduction (main text) reflects the fact that OV is not an independent life history axis in mammals and conversely that these results reflect the associations of OV with the life history strategy of a species.

**Table S3.** Pairwise phylogenetic generalized linear model between offspring value index (OV) and each life history trait (LH) at each stage of invasion. We report the mean and SD of posterior distribution of *β* estimates for the slope of the association between OV and each life history trait, and the percentage of *β* posterior distribution crossing over 0. Variable names: BM (adult body mass), GT (gestation time), WA (weaning age), NBM (neonatal body mass), LS (litter size), LY (litters per year), AFB (age at first birth), RL (reproductive lifespan).

| Stage | Introduction | | | Establishment | |  | Spread | |  |
| --- | --- | --- | --- | --- | --- | --- | --- | --- | --- |
| LH | Mean *β* | SD *β* | % *β* | Mean *β* | SD *β* | % *β* | Mean *β* | SD *β* | % *β* |
| BM | -0.05 | 0.02 | 0.0 | -0.08 | 0.03 | 0.3 | -0.13 | 0.04 | 0.0 |
| GT | -0.12 | 0.07 | 5.0 | -0.30 | 0.10 | 0.2 | -0.47 | 0.17 | 0.3 |
| WA | 0.02 | 0.04 | 30.8 | -0.01 | 0.08 | 44.1 | -0.18 | 0.12 | 6.0 |
| NBM | -0.08 | 0.02 | 0.0 | -0.11 | 0.03 | 0.1 | -0.16 | 0.04 | 0.0 |
| LS | 0.26 | 0.07 | 0.0 | 0.41 | 0.10 | 0.0 | 0.31 | 0.18 | 4.2 |
| LY | -0.79 | 0.06 | 0.0 | -0.87 | 0.11 | 0.0 | -0.66 | 0.17 | 0.0 |
| AFB | 0.01 | 0.04 | 37.6 | 0.10 | 0.08 | 9.8 | -0.02 | 0.10 | 42.8 |
| RL | -0.89 | 0.04 | 0.0 | -0.93 | 0.08 | 0.0 | -0.85 | 0.08 | 0.0 |

2.3 – Potential sampling biases

We assess whether there are biases in the total pool of species used in the main analysis (n=518) and compare this pool (‘Included’ species, see main text) with mammals for which at least one life history trait is missing (‘Excluded’ species sample; sections 1.1.4). The phylogenetic t-tests show that there are small biases in the sample of species for which we have all available life history data. Species included in our dataset for the main analysis (‘Included’ sample) differ from the ‘Excluded’ species in three life history traits: body mass, litter size, and reproductive lifespan (Table S4). However, the *β* values for such differences are small, particularly when considered in the light of the large sample sizes of this analysis (Table S4).

**Table S4.** Phylogenetic t-test in PGLS on the mean values of each life history trait between the sample of species used in this study (‘Included’, n=518, main text) and those excluded (‘Excluded’, section 1.2.4). The sample size of the species excluded from our analysis (‘Excluded’) varies for each life history trait tested. Here we report the total sample size for this analysis (included and excluded species together, ‘N total’), the sample size of the ‘Excluded’ sample (‘N Excluded’), and the t-value, p-value and strength of the phylogenetic signal, as quantified by the λ parameter in PGLS, for the comparison between the species included and those excluded (see section 1.2.7). Variable names: BM (adult body mass), GT (gestation time), WA (weaning age), NBM (neonatal body mass), LS (litter size), LY (litters per year), AFB (age at first birth), RL (reproductive lifespan).

| Life history trait | N total | N Excluded | β | T | P | λ |
| --- | --- | --- | --- | --- | --- | --- |
| BM | 3506 | 2989 | 0.06 | 4.5 | <0.001 | 0.99 |
| GT | 1427 | 909 | <0.01 | 0.1 | 0.923 | 0.99 |
| WA | 1252 | 734 | <0.01 | 0.3 | 0.737 | 0.92 |
| NBM | 1107 | 589 | <0.01 | 0.2 | 0.844 | 0.99 |
| LS | 2536 | 2018 | 0.03 | 5.8 | <0.001 | 0.96 |
| LY | 1230 | 712 | 0.01 | 1.0 | 0.307 | 0.93 |
| AFB | 944 | 426 | 0.02 | 1.7 | 0.096 | 0.90 |
| RL | 748 | 230 | 0.09 | 6.0 | <0.001 | 0.93 |

2.4 – Alternative transformations for introduction effort

Introduction effort is highly skewed across species since most mammals have been introduced to one or few locations (main text, section 1.1.2). Following previous studies (Duncan *et al.* 2001; Forsyth *et al.* 2004; Krivánek *et al.* 2006; van Wilgen & Richardson 2012), we convert introduction effort into a binary variable with a split at 4, the median number of introduction locations for all introduced species with data on establishment success (section 1.2.1), for the analysis in the main text and supporting analyses so far presented (sections 2.1, 2.3). Here we show that the main conclusions of this study remained unaltered once alternative thresholds to convert introduction effort into a binary variable, or the raw data or the log-transformed data, are used.

Consistent with the main analysis (median split at 4 location, main text) we repeatedly find that, at establishment, litter size and introduction effort are strongly associated with success regardless how introduction effort is analysed (Table S5a). Likewise, reproductive lifespan has a weaker influence on success at this stage and its effect becomes stronger with a higher threshold for the conversion of introduction effort into a binary trait (Table S5a). Moreover, no other life history trait is associated with success at this stage in any model (Table S5a).

At the stage of spread, litter size and introduction effort are strongly associated with success in all models while litters per year has a weaker effect across all models (Table S5b), as found for the main analysis (main text). No other life history trait is associated with success at this stage with the sole exceptions of marginally older age at first birth in successful mammals in the model with introduction effort split at 6 locations and an older weaning age for successful mammals with introduction effort split at 3 locations (Table S5b). However, both results are not robust and are not associated with success in any other model regardless of how introduction effort is analysed (Table S5b). In addition, we note that a small number of species fall in the category ‘unsuccessful’ (n=13 out of 74 species at this stage) when introduction effort is split at 3 at this invasion stage (Table S5b).

**Table S5.** Phylogenetic GLMM models of success at establishment (a) and spread (b) against all life history traits and introduction effort when introduction effort (IE) is analysed as raw data, log-transformed data or is transformed into a binary variable using different thresholds (establishment stage: n=149; spread stage: n=74; see Figure 1a in the main text). The column labeled ‘Transform’ indicates how IE is treated and the next column how many species falls in the category ‘0’ for IE treated as binary (N [0]). The median split at 4 is used in the main analysis (0 as ≤4, 1 as ≥5). The remaining columns report the mean *beta* estimates and proportion of overlap with 0 for each variable in the model (section 1.2.3). Variable names: BM (adult body mass), GT (gestation time), WA (weaning age), NBM (neonatal body mass), LS (litter size), LY (litters per year), AFB (age at first birth), RL (reproductive lifespan).

| (a) Establishment | | IE |  | BM |  | GT |  | WA |  | NBM |  | LS |  | LY |  | AFB |  | RL |  |
| --- | --- | --- | --- | --- | --- | --- | --- | --- | --- | --- | --- | --- | --- | --- | --- | --- | --- | --- | --- |
| Transform | N [0] | β | % | β | % | β | % | β | % | β | % | β | % | β | % | β | % | β | % |
| Raw data | NA | 0.34 | 0.0 | -0.40 | 32.0 | -1.91 | 21.0 | 0.57 | 33.9 | 0.08 | 47.2 | 5.27 | 2.2 | 1.31 | 28.7 | 0.07 | 48.4 | 3.32 | 6.2 |
| Log_10_ | NA | 3.61 | 0.0 | -0.39 | 32.1 | -2.22 | 18.2 | 0.42 | 38.5 | 0.17 | 43.2 | 4.45 | 4.4 | 1.51 | 24.8 | 0.13 | 46.7 | 2.71 | 9.4 |
| ≤3, ≥4 | 64 | 3.07 | 0.0 | -0.19 | 40.8 | -1.67 | 23.9 | 0.89 | 27.1 | 0.05 | 48.4 | 5.46 | 1.2 | 1.36 | 28.0 | 0.26 | 43.7 | 2.26 | 12.2 |
| ≤4, ≥5 | 71 | 2.70 | <0.1 | -0.30 | 36.0 | -2.20 | 17.5 | 0.90 | 26.0 | 0.15 | 42.9 | 5.51 | 0.9 | 1.44 | 25.9 | 0.61 | 34.9 | 2.49 | 8.9 |
| ≤5, ≥6 | 81 | 2.10 | <0.1 | -0.17 | 42.0 | -1.88 | 20.6 | 0.80 | 28.3 | 0.05 | 47.6 | 5.96 | 0.7 | 1.93 | 17.7 | 0.39 | 40.4 | 2.91 | 5.2 |
| ≤6, ≥7 | 89 | 2.43 | <0.1 | -0.24 | 37.9 | -2.07 | 18.5 | 0.76 | 29.6 | 0.13 | 44.3 | 6.17 | 0.4 | 1.84 | 18.4 | 0.47 | 37.1 | 2.95 | 5.5 |
| ≤7, ≥8 | 93 | 2.85 | 0.0 | -0.31 | 34.2 | -1.86 | 21.5 | 0.99 | 23.9 | 0.08 | 46.4 | 5.68 | 0.7 | 1.34 | 26.6 | -0.21 | 44.0 | 3.20 | 4.3 |
| ≤8, ≥9 | 96 | 2.73 | <0.1 | -0.31 | 35.1 | -1.74 | 22.6 | 1.04 | 22.5 | 0.05 | 47.7 | 5.59 | 0.6 | 1.36 | 25.7 | -0.45 | 37.8 | 3.27 | 3.2 |
| ≤9, ≥10 | 101 | 2.50 | <0.1 | -0.25 | 37.3 | -1.79 | 21.1 | 0.83 | 27.3 | 0.06 | 47.4 | 5.67 | 0.5 | 1.51 | 22.9 | -0.41 | 38.9 | 3.33 | 3.1 |

| (b) Spread | | IE |  | BM |  | GT |  | WA |  | NBM |  | LS |  | LY |  | AFB |  | RL |  |
| --- | --- | --- | --- | --- | --- | --- | --- | --- | --- | --- | --- | --- | --- | --- | --- | --- | --- | --- | --- |
| Transform | N [0] | β | % | β | % | β | % | β | % | β | % | β | % | β | % | β | % | β | % |
| Raw data | NA | 0.04 | <0.1 | 0.61 | 21.5 | 0.99 | 36.8 | 0.81 | 33.2 | -0.67 | 25.4 | 3.56 | 5.1 | 2.47 | 12.9 | 0.58 | 35.3 | 1.09 | 28.8 |
| Log_10_ | NA | 2.14 | 0.0 | 0.19 | 40.1 | 1.21 | 36.4 | 0.91 | 30.7 | -0.61 | 29.0 | 3.34 | 7.2 | 2.82 | 12.4 | 1.23 | 20.4 | 0.65 | 36.6 |
| ≤3, ≥4 | 13 | 2.82 | <0.1 | 0.16 | 42.0 | -0.77 | 42.3 | 2.57 | 8.9 | <0.01 | 49.8 | 4.78 | 2.2 | 3.71 | 6.2 | 1.30 | 20.6 | 0.26 | 44.6 |
| ≤4, ≥5 | 17 | 1.77 | 1.0 | 0.28 | 36.3 | -0.54 | 43.1 | 1.96 | 13.8 | -0.23 | 42.5 | 3.93 | 3.7 | 3.15 | 8.2 | 1.55 | 15.4 | 0.31 | 43.1 |
| ≤5, ≥6 | 19 | 1.85 | 0.8 | 0.28 | 37.0 | -0.23 | 47.3 | 1.65 | 18.3 | -0.37 | 37.4 | 3.86 | 4.1 | 3.54 | 5.5 | 1.85 | 11.0 | 0.76 | 34.5 |
| ≤6, ≥7 | 25 | 3.21 | 0.0 | 0.34 | 34.4 | 0.71 | 42.0 | 1.42 | 23.4 | -0.70 | 27.3 | 4.40 | 2.7 | 3.65 | 7.5 | 2.54 | 5.7 | -0.08 | 47.8 |
| ≤7, ≥8 | 27 | 3.36 | 0.0 | 0.12 | 43.9 | 0.12 | 48.5 | 2.03 | 15.9 | -0.39 | 37.1 | 4.04 | 4.2 | 2.82 | 13.8 | 1.68 | 14.5 | -0.05 | 47.7 |
| ≤8, ≥9 | 28 | 2.90 | 0.0 | 0.17 | 41.3 | 0.95 | 38.9 | 1.52 | 21.1 | -0.57 | 31.3 | 3.39 | 6.5 | 2.80 | 13.6 | 0.92 | 26.9 | 0.41 | 41.9 |
| ≤9, ≥10 | 32 | 2.23 | <0.1 | 0.30 | 35.5 | 0.72 | 41.7 | 1.31 | 23.2 | -0.52 | 32.6 | 3.75 | 4.6 | 3.34 | 8.6 | 1.04 | 25.0 | 0.82 | 33.5 |
| ≤10, ≥11 | 34 | 2.18 | <0.1 | 0.37 | 31.5 | 0.37 | 45.5 | 1.16 | 26.0 | -0.45 | 34.5 | 3.59 | 4.2 | 3.54 | 7.0 | 0.92 | 27.4 | 0.69 | 35.4 |

3 – SUPPLEMENTARY DISCUSSION

The analyses presented here show that the main conclusion of this study on how life history traits affect each stage of the invasion pathway are robust. Results do not differ qualitatively once possible issues with multicollinearity among predictors are considered (section 2.1). Furthermore, our results and conclusions are unlikely to be affected by potential biases associated with the sample of mammals included in our study (see section 2.3) and by how introduction effort is analysed (section 2.4).

Specifically, our conclusions are consistent between full and reduced models after multicollinearity is considered (section 2.1). The probability of being introduced into novel environments is higher for species with larger and more frequent litters and a longer reproductive lifespan, as found in the main analysis (sections 2.1). In support of classic theory on how life history traits enable small populations to grow and escape the risk of extinction due to stochasticity (Pimm 1991), we find that the probability of establishing an alien population is higher for introduced species with larger litters but, consistent with recent theoretical models (Sæther *et al.* 2004; Jeppsson & Forslund 2012), in conjunction with a weaker contribution of longer reproductive lifespan (section 2.1). Finally, our finding that established mammals are more likely to spread in non-native regions if they have larger litters and, to a lesser extent, more frequent litters, is consistent between full and reduced models (section 2.1). At spread, we also find a small positive effect on weaning age on the probability of success when possible issues with multicollinearity are resolved (section 2.1). Success at both the establishment and spread stages increases with greater introduction effort in the reduced models (section 2.1), as found in the main analysis (main text). Our results also indicate that OV is not an independent life history axis in mammals (section 2.2).

Sampling biases are unlikely to affect our conclusions (section 2.4). Influential variables in the main analysis that show slight sampling biases are litter size and reproductive lifespan. However the differences between the species included and excluded in the main analysis are small for such large samples. Finally, the way introduction effort is treated for the analysis (as raw data, as log-transformed, or as binary with different thresholds; see section 1.1.1) does not influence our conclusions on which life history traits are influential for success at establishment and spread (section 2.4). Regardless of how introduction effort is analysed, establishment success is higher for mammals with larger litters and marginally longer lifespan, while success at spread is associated with larger litters and, more weakly, with more frequent litters. At both stages, greater introduction effort is associated with success.

Finally, our approach demonstrates the importance of estimating and accounting for the phylogenetic signal in the data when testing hypotheses across species on factors promoting success of alien organisms. In all models at all stages of invasion we find that the strength of the phylogenetic signal is intermediate between 0 (species’ independence) and 1 (similarity between species is proportional to the time of common evolution; Freckleton *et al.* 2002). These results indicate that methods assuming that phylogenetic effects are very high (e.g. independent contrasts) or non-existent (i.e. without phylogeny), are inappropriate and might lead to incorrect conclusions in studies of biological invasions that underestimate or overestimate the strength of phylogenetic signal. It should be noted that statistical models that include phylogenies evaluate the strength of the phylogenetic signal on latent variables (e.g. GLMM in this study; Hadfield 2010; Hadfield & Nakagawa 2010; Hadfield 2012) or model residuals (e.g. PGLS; Freckleton *et al.* 2002; Revell 2010). Therefore, such estimates of phylogenetic signal are based on all available data in a model (in our case invasion success and all predictors) and cannot be used in a simple fashion for inferring whether closely related species are more or less likely to succeed at different stages than less closely related species.

Altogether this study supports classic theory (Pimm 1991) regarding the role of life history traits in sustaining population growth and demonstrates that along the invasion pathway barriers at each stage select for species with progressively greater reproductive output; invasive mammals are therefore very fecund.

Our approach of studying invasion at the species level is particularly suited for testing hypotheses on which species traits, as opposed to the location or community characteristics, promote success of alien organisms (see Section 1.1.2), while also effectively accounting for phylogenetic effects across species. We also demonstrate that the number of unique introduction locations can be used as a robust and reliable estimate of the magnitude of introduction effort in comparative studies across species (Sections 1.1.3 and 2.4). We thus argue that the combination of a species level approach like ours, our detailed protocol for the classification of success at each stage along the invasion pathway from introduction to spread, and the number of introduction locations as an estimate of introduction effort, will be particularly valuable to investigate which species traits promote invasion success in taxa where detailed data on the number of introduced individuals is unknown or is available for too few introductions.

4 – SUPPLEMENTARY REFERENCES

4.1 – Supplementary references

1.Bielby, J., Mace, G., Bininda Emonds, O., Cardillo, M., Gittleman, J., Jones, K., *et al.* (2007). The fast‐slow continuum in mammalian life history: an empirical reevaluation. *Am. Nat.*, 169, 748–757.

2.Blackburn, T.M., Pyšek, P., Bacher, S., Carlton, J.T., Duncan, R.P., Jarošík, V., *et al.* (2011). A proposed unified framework for biological invasions. *TREE*, 26, 333–339.

3.Bomford, M., Kraus, F., Barry, S.C. & Lawrence, E. (2009). Predicting establishment success for alien reptiles and amphibians: a role for climate matching. *Biol Invasions*, 11, 713–724.

4.Bókony, V., Lendvai, Á.Z., Liker, A., Angelier, F., Wingfield, J.C. & Chastel, O. (2009). Stress Response and the Value of Reproduction: Are Birds Prudent Parents? *Am. Nat.*, 173, 589–598.

5.Carey, J.R. & Judge, D.S. (2000). *Longevity records. Life spans of mammals, birds, amphibians, reptiles and fish*. Odense University Press, Odense.

6.Cassey, P., Blackburn, T.M., Sol, D., Duncan, R.P. & Lockwood, J.L. (2004). Global patterns of introduction effort and establishment success in birds. *Proc R Soc B*, 271, S405–S408.

7.DAISIE. (2008). *Handbook of Alien Species in Europe*. Springer.

8.De Magalhaes, J.P. & Costa, J. (2009). A database of vertebrate longevity records and their relation to other life-history traits. *J. Evol. Biol.*, 22, 1770–1774.

9.Duncan, R.P., Bomford, M., Forsyth, D.M. & Conibear, L. (2001). High predictability in introduction outcomes and the geographical range size of introduced Australian birds: a role for climate. *J Anim Ecol*, 70, 621–632.

10.Ernest, S.K.M. (2003). Life History Characteristics of Placental Nonvolant Mammals: Ecological Archives E084-093. *Ecology*, 84.

11.Forsyth, D.M. & Duncan, R.P. (2001). Propagule Size and the Relative Success of Exotic Ungulate and Bird Introductions to New Zealand. *Am. Nat.*, 157, 583–595.

12.Forsyth, D.M., Duncan, R.P., Bomford, M. & Moore, G. (2004). Climatic Suitability, Life‐History Traits, Introduction Effort, and the Establishment and Spread of Introduced Mammals in Australia. *Conserv Biol*, 18, 557–569.

13.Freckleton, R., Harvey, P. & Pagel, M. (2002). Phylogenetic analysis and comparative data: a test and review of evidence. *Am. Nat.*, 160, 712–726.

14.Greene, W.H. (2012). *Econometric analysis*. 7 edn. Pearson Education, Harlow.

15.Hadfield, J.D. (2010). MCMC methods for multi-response generalized linear mixed models: the MCMCglmm R package. *J Stats Softw*, 33, 1–22.

16.Hadfield, J.D. (2012). MCMCglmm Course Notes, 1–138.

17.Hadfield, J.D. & Nakagawa, S. (2010). General quantitative genetic methods for comparative biology: phylogenies, taxonomies and multi-trait models for continuous and categorical characters. *J. Evol. Biol.*, 23, 494–508.

18.IUCN (Ed.). (2013). *Global Invasive Species Database (GISD)*. URL http://www.issg.org/database/welcome/.

19.Ives, A.R., Midford, P.E. & Garland, T., JR. (2007). Within-Species Variation and Measurement Error in Phylogenetic Comparative Methods. *Syst Biol*, 56, 252–270.

20.Jeppsson, T. & Forslund, P. (2012). Can life history predict the effect of demographic stochasticity on extinction risk? *Am. Nat.*, 179, 706–720.

21.Jones, K.E., Bielby, J., Cardillo, M., Fritz, S.A., O'Dell, J., Orme, C.D.L., *et al.* (2009). PanTHERIA: a species-level database of life history, ecology, and geography of extant and recently extinct mammals. *Ecology*, 90, 2648–2648.

22.Kolar, C.S. & Lodge, D.M. (2001). Progress in invasion biology: predicting invaders. *TREE*, 16, 199–204.

23.Krivánek, M., Pyšek, P. & Jarošík, V. (2006). Planting history and propagule pressure as predictors of invasion by woody species in a temperate region. *Conserv Biol*, 20, 1487–1498.

24.Lockwood, J.L., Cassey, P. & Blackburn, T. (2005). The role of propagule pressure in explaining species invasions. *TREE*, 20, 223–228.

25.Long, J.L. (2003). *Introduced Mammals of the World: Their History, Distribution and Influence*. CABI Publishing.

26.Long, J.S. (1997). *Regression models for categorical and limited dependent variables*. SAGE Publications, Inc., London.

27.Marchetti, M.P., Moyle, P.B. & Levine, R. (2004). Invasive species profiling? Exploring the characteristics of non‐native fishes across invasion stages in California. *Freshwater Biol*, 49, 646–661.

28.Mood, C. (2010). Logistic Regression: Why We Cannot Do What We Think We Can Do, and What We Can Do About It. *European Sociological Review*, 26, 67–82.

29.Pagel, M., Meade, A. & Barker, D. (2004). Bayesian Estimation of Ancestral Character States on Phylogenies. *Syst Biol*, 53, 673–684.

30.Pimm, S. (1991). *The balance of nature?* University of Chicago Press, Chicago.

31.Quinn, G. & Keough, M. (2007). *Experimental design and data analysis for biologists*. 6 edn. Cambridge University Press, Cambridge.

32.Rago, A., While, G.M. & Uller, T. (2012). Introduction pathway and climate trump ecology and life history as predictors of establishment success in alien frogs and toads. *Ecology and Evolution*, 2, 1437–1445.

33.Revell, L.J. (2010). Phylogenetic signal and linear regression on species data. *Methods Ecol Evol*, 1, 319–329.

34.Revell, L.J. & Graham Reynolds, R. (2012). A new Bayesian method for fitting evolutionary models to comparative data with intraspecific variation. *Evolution*, 66, 2697–2707.

35.Richardson, D.M. & Pyšek, P. (2012). Naturalization of introduced plants: ecological drivers of biogeographical patterns. *New Phytologist*, 196, 383–396.

36.Simberloff, D. (2009). The role of propagule pressure in biological invasions. *Annu. Rev. Ecol. Evol. Syst.*, 40, 81–102.

37.Sol, D., Maspons, J., Vall-Llosera, M., Bartomeus, I., García-Peña, G.E., Piñol, J., *et al.* (2012). Unraveling the life history of successful invaders. *Science*, 337, 580–583.

38.Sæther, B.E., Engen, S., Pape Møller, A., Weimerskirch, H., Visser, M.E., Fiedler, W., *et al.* (2004). Life history variation predicts the effects of demographic stochasticity on avian population dynamics. *Am. Nat.*, 164, 793–802.

39.Tingley, R., Phillips, B.L. & Shine, R. (2011). Establishment Success of Introduced Amphibians Increases in the Presence of Congeneric Species. *Am. Nat.*, 177, 382–388.

40.van Kleunen, M., Dawson, W., Schlaepfer, D., Jeschke, J.M. & Fischer, M. (2010a). Are invaders different? A conceptual framework of comparative approaches for assessing determinants of invasiveness. *Ecol Lett*, 13, 947–958.

41.van Kleunen, M., Weber, E. & Fischer, M. (2010b). A meta-analysis of trait differences between invasive and non-invasive plant species. *Ecol Lett*, 13, 235–245.

42.van Wilgen, N.J. & Richardson, D.M. (2012). The roles of climate, phylogenetic relatedness, introduction effort, and reproductive traits in the establishment of non-native reptiles and amphibians. *Conserv Biol*, 26, 267–277.

43.Wilson, D.E. & Reeder, D.M. (Eds.). (2005). *Mammal Species of the World. A Taxonomic and Geographic Reference*. 3rd edn. Johns Hopkins University Press, Baltimore, Maryland (USA).

4.2 – Supplementary data references

1.Álvarez-Castañeda, S.T., Arnaud, G., Cortés-Calva, P. & Méndez, L. (2010). Invasive migration of a mainland rodent to santa catalina island and its effect on the endemic species Peromyscus slevini. *Biol. Invasions*, 12, 437–439.

2.Atlas of Irish Mammals [WWW Document]. (2011). *Natl. Biodivers. Data Cent.* URL http://mammals.biodiversityireland.ie/.

3.Bennett, M.D. (2008). Western barred bandicoots in health and disease. PhD Thesis. Murdoch University.

4.Boye, P. (1991). Notes on the morphology, ecology and geographic origin of the Cyprus long-eared hedgehog (Hemiechinus auritus dorotheae). *Bonner Zool. Beiträge*, 42, 115–123.

5.British Isles Exotic and Introduced Mammals [WWW Document]. (2011). *Mark Hows* . URL http://www.hows.org.uk/inter/birds/exotics/gbm.htm.

6.Broekhuizen, S. & Maaskamp, F. (1980). Behaviour of does and leverets of the European hare (Lepus europaeus) whilst nursing. *J. Zool.*, 191, 487–501.

7.Buckner, C. (1966). Populations and Ecological Relationships of Shrews in Tamarack Bogs of Southeastern Manitoba. *J. Mammal.*, 47, 181–194.

8.Buzas, B. & Gulyas, E. (2012). Hand-raising jaguarundis. *Feline Conserv. Fed.*, 20–22.

9.Campos, P.F., Willerslev, E., Sher, A., Orlando, L., Axelsson, E., Tikhonov, A., *et al.* (2010). Ancient DNA analyses exclude humans as the driving force behind late Pleistocene musk ox (*Ovibos moschatus*) population dynamics. *Proc. Natl. Acad. Sci. U. S. A.*, 107, 5675–5680.

10.Capellini, I., Venditti, C. & Barton, R.A. (2011). Placentation and maternal investment in mammals. *Am. Nat.*, 177, 86–98.

11.Cervus duvaucelii [WWW Document]. (2013). *Encycl. Life*. URL http://eol.org/pages/308523/overview.

12.Van Dyck, S. & Strahan, R. (Eds.). (2008). *The mammals of Australia*. Holland Pub Pty Ltd., Sydney, Australia.

13.Estrada, A., Estrada, R. & Ervin, F. (1977). Establishment of a free-ranging colony of stumptail macaques (Macaca arctoides): social relations I. *Primates*, 18, 647–676.

14.Fautley, R., Coulson, T. & Savolainen, V. (2012). A comparative analysis of the factors promoting deer invasion. *Biol. Invasions*, 14, 2271–2281.

15.Feldhamer, G., Thompson, B. & Chapman, J. (Eds.). (n.d.). *Wild mammals of North America*. 2nd edn. John Hopkins’ University Press, Baltimore, MD.

16.Gerbils on the Isle of Wight [WWW Document]. (2011). *eGerbil*. URL http://www.egerbil.com/iow.html.

17.Gittleman, J.L. (1986). The American Society of Naturalists Carnivore Life History Patterns : Allometric , Phylogenetic , and Ecological Associations. *Am. Nat.*, 127, 744–771.

18.Gliwicz, J. & Taylor, J.R.E. (2002). Comparing life histories of shrews and rodents. *Acta Theriol. (Warsz).*

19.Gottdenker, N. & Bodmer, R.E. (1998). Reproduction and productivity of white-lipped and collared peccaries in the Peruvian Amazon. *J. Zool.*, 245, 423–430.

20.Gustafson, L.L., Franklin, W.L., Sarno, R.J., Hunter, R.L., Young, K.M., Johnson, W.E., *et al.* (1998). Predicting Early Mortality of Newborn Guanacos by Birth Mass and Hematological Parameters: A Provisional Model. *J. Wildl. Manage.*, 62, 24–35.

21.Hansen, B.K., Jeppesen, L.L. & Berg, P. (2010). Stereotypic behaviour in farm mink (Neovison vison) can be reduced by selection. *J. Anim. Breed. Genet.*, 127, 64–73.

22.Hulley, J.T. (1976). Maintenance and breeding of captive jaguarundis at Chester Zoo and Toronto. *Int. Zoo Yearb.*, 16, 120–122.

23.Hunt, A. (2011). Panthera pardus [WWW Document]. *Anim. Divers. Web*. URL http://animaldiversity.org/accounts/Panthera_pardus/.

24.Kingdon, J., Happold, D., Butynski, T.M., Hoffmann, M., Happold, M. & Kalina, J. (Eds.). (2013). *Mammals of Africa (6 vols)*. Bloomsbury Publishing, London.

25.Langer, P. (2002). The digestive tract and life history of small mammals. *Mamm. Rev.*, 32, 107–131.

26.Lever, C. (1985). *Naturalized mammals of the World*. Longman, London.

27.Lever, C. (2009). *The naturalized animals of Britain and Ireland*. New Holland.

28.Mallia, E., Rugge, C., Cosentino, C., Gambacorta, E., Trocchi, V. & Freschi, P. (2010). Postnatal growth of Brown hare (Lepus europaeus) in a South Italy rearing centre. *Ital. J. Anim. Sci.*, 8.

29.McDevitt, A.D., Rambau, R. V., O’Brien, J., McDevitt, C.D., Hayden, T.J. & Searle, J.B. (2009). Genetic variation in Irish pygmy shrews Sorex minutus (Soricomorpha: Soricidae): Implications for colonization history. *Biol. J. Linn. Soc.*, 97, 918–927.

30.Miyazaki, N. (2001). A review of studies on the ringed, Caspian, and Baikal seals: Pusa hispida, P. caspica, and P. sibirica. *Otsuchi Mar. Sci.*, 26, 1–6.

31.Nature in Shetland [WWW Document]. (2014) . URL http://www.nature-shetland.co.uk/.

32.No Striped Skunk [WWW Document]. (2014). *Dep. Agric. For.* URL http://www.gov.pe.ca/forestry/index.php3?number=1010080.

33.Nonnatives - Squirrell Monkey [WWW Document]. (2014). *Florida Fish Wildl. Conserv. Comm.* URL http://myfwc.com/wildlifehabitats/nonnatives/mammals/squirrel-monkey/.

34.O’Keeffe, R.T., Lifshitz, K. & Linn, G. (1983). Relationships among dominance, inter-animal spatial proximity and affiliative social behavior in stumptail macaques (Macaca arctoides). *Appl. Anim. Ethol.*

35.Olson, K.A., Fuller, T.K., Schaller, G.B., Lhagvasuren, B. & Odonkhuu, D. (2005). Reproduction, neonatal weights, and first-year survival of Mongolian gazelles (Procapra gutturosa). *J. Zool.*, 265, 227–233.

36.Özkan, B. (2006). An observation on the reproductive biology of Glis glis (Linnaeus, 1766)(Rodentia; Gliridae) and body weight gaining of pups in the Istranca Mountains of Turkish Thrace. *Int. J. Zool. Res.*, 2.

37.Page, A., Kirkpatrick, W. & Massam, M. (2009). *Risk Assessment for Australia*. Government of Western Australia.

38.Pietri, C., Alves, P.C. & Melo-Ferreira, J. (2011). Hares in Corsica: High prevalence of Lepus corsicanus and hybridization with introduced L. europaeus and L. granatensis. *Eur. J. Wildl. Res.*, 57, 313–321.

39.Purwantara, B., Noor, R.R., Andersson, G. & Rodriguez-Martinez, H. (2012). Banteng and Bali cattle in Indonesia: Status and forecasts. *Reprod. Domest. Anim.*, 47, 2–6.

40.Putnam, R. (1988). *The natural history of deer*. Cornell University Press.

41.Råberg, L., Loman, J., Hellgren, O., van der Kooij, J., Isaksen, K. & Solheim, R. (2013). The origin of Swedish and Norwegian populations of the Eurasian harvest mouse (Micromys minutus). *Acta Theriol. (Warsz).*, 58, 101–104.

42.Reynolds, J.C., O’Mahony, D. & Aebischer, N.J. (2006). Implications of “cyclical” population dynamics for the conservation of Irish hares (Lepus timidus hibernicus). *J. Zool.*, 270, 408–413.

43.Richards, J.D. & Short, J. (2003). Reintroduction and establishment of the western barred bandicoot Perameles bougainville (Marsupialia: Peramelidae) at Shark Bay, Western Australia. *Biol. Conserv.*, 109, 181–195.

44.Richardson, K. (2012). *Australia’s Amazing Kangaroos*. CSIRO Publishing, Collingwood.

45.Rismiller, P.D. & McKelvey, M.W. (2003). Body mass, age and sexual maturity in short-beaked echidnas, Tachyglossus aculeatus. In: *Comp. Biochem. Physiol. - A Mol. Integr. Physiol.* pp. 851–865.

46.Robinson, J. & Bennett, E. (Eds.). (2000). *Hunting for sustainability in tropical forests*. Columbia University Press, New York.

47.Robinson, R. (1977). *Genetics for cat breeders*. 2nd edn. Pergamon Press, Oxford, England.

48.Rubondo Island National Park [WWW Document]. (2014). *Tanzania Parks Authority.* URL http://www.tanzaniaparks.com/rubondo.html.

49.Say, L., Pontier, D. & Natoli, E. (2001). Influence of oestrus synchronization on male reproductive success in the domestic cat (Felis catus L.). *Proc. Biol. Sci.*, 268, 1049–1053.

50.Schuster, R.H. (1976). Lekking behavior in kafue lechwe. *Science*, 192, 1240–1242.

51.Semiadi, G., Barry, T.N. & Muir, P.D. (1993). Growth, milk intake and behaviour of artificially reared sambar deer (Cervus unicolor) and red deer (Cervus elaphus) fawns. *J. Agric. Sci.*, 121, 273.

52.Sidorovich, V.A.D.I.M. (2001). Study on the decline in the European mink Mustela lutreola population in connection with the American mink M. vison expansion in Belarus: story of the study, review of the results and research priorities. *Säugetierkundliche Informationen*, 5, 133–153.

53.Simmons, N.M., Bayer, M.B. & Sinkey, L.O. (1984). Demography of Dall’s sheep in the Mackenzie Mountains, Northwest Territories. *J. Wildl. Manage.*, 48, 156–162.

54.SNH renews stoat appeal [WWW Document]. (2013). The Orcadian. URL https://www.orcadian.co.uk/2013/07/snh-renew-stoat-appeal/.

55.Stefan, C.I. & Krebs, C.J. (2001). Reproductive changes in a cyclic population of snowshoe hares. *Can. J. Zool.*

56.Taggart, D.A., Schultz, D., White, C., Whitehead, P., Underwood, G. & Phillips, K. (2005). Cross-fostering, growth and reproductive studies in the brush-tailed rock-wallaby, Petrogale penicillata (Marsupialia:Macropodidae): Efforts to accelerate breeding in a threatened marsupial species. *Aust. J. Zool.*, 53, 313–323.

57.Tanner, Z. & Hocking, G. (2001). *Status and Management of the Forester Kangaroo in Tasmania*. Nature Conservation Branch, Dept. of PRimary Industries, Water and Environment, Hobart.

58.Vasilieva, N.A. & Tchabovsky, A. V. (2014). Timing is the only thing: reproduction in female yellow ground squirrels (Spermophilus fulvus). *Can. J. Zool.*, 92, 737–747.

59.Vidus-Rosin, A., Meriggi, A., Cardarelli, E., Serrano-Perez, S., Mariani, M.C., Corradelli, C., *et al.* (2010). Habitat overlap between sympatric European hares (Lepus europaeus) and Eastern cottontails (Sylvilagus floridanus) in northern Italy. *Acta Theriol. (Warsz).*, 56, 53–61.

60.Wenying, C., Zhengdong, N., Tinglin, W. & Bo, Z. (1997). Growth and Development of Siberian chipmunk (Eutamias sibiricus). *J. Shaanxi Norm. Univ.*, 1.

61.Wright, L.J., Newson, S.E. & Noble, D.G. (2014). The value of a random sampling design for annual monitoring of national populations of larger British terrestrial mammals. *Eur. J. Wildl. Res.*, 60, 213–221.

62.Wu, H. & Lin, Y. (1992). Life history variables of wild troops of formosan macaques (Macaca cyclopis) in Kenting, Taiwan. *Primates*, 33, 85–97.
